# Defective angiogenesis in CXCL12 mutant mice impairs skeletal muscle regeneration

**DOI:** 10.1186/s13395-019-0210-5

**Published:** 2019-09-18

**Authors:** David Hardy, Mylène Fefeu, Aurore Besnard, David Briand, Paméla Gasse, Fernando Arenzana-Seisdedos, Pierre Rocheteau, Fabrice Chrétien

**Affiliations:** 1 Experimental Neuropathology Unit, Institut Pasteur, 75015 Paris, France; 2 Viral Pathogenesis Unit, Institut Pasteur, 75015 Paris, France; 3 Service Hospitalo-Universitaire de Psychiatrie, Centre Hospitalier Sainte Anne, 75014 Paris, France; 4 Paris Descartes University, Sorbonne Paris Cité, 75006 Paris, France; 5 Service Hospitalo-Universitaire de Neuropathologie, Centre Hospitalier Sainte Anne, 75014 Paris, France

**Keywords:** Muscle stem cells, Endothelial cells, CXCL12, Heparan sulfates, Skeletal muscle, Regeneration, Angiogenesis, Vasculogenesis, Fibrosis

## Abstract

**Background:**

During muscle regeneration, the chemokine CXCL12 (SDF-1) and the synthesis of some specific heparan sulfates (HS) have been shown to be critical. CXCL12 activity has been shown to be heavily influenced by its binding to extracellular glycosaminoglycans (GAG) by modulating its presentation to its receptors and by generating haptotactic gradients. Although CXCL12 has been implicated in several phases of tissue repair, the influence of GAG binding under HS influencing conditions such as acute tissue destruction remains understudied.

**Methods:**

To investigate the role of the CXCL12/HS proteoglycan interactions in the pathophysiology of muscle regeneration, we performed two models of muscle injuries (notexin and freeze injury) in mutant CXCL12^Gagtm/Gagtm^ mice, where the CXCL12 gene having been selectively mutated in critical binding sites of CXCL12 to interact with HS. Histological, cytometric, functional transcriptomic, and ultrastructure analysis focusing on the satellite cell behavior and the vessels were conducted on muscles before and after injuries. Unless specified, statistical analysis was performed with the Mann-Whitney test.

**Results:**

We showed that despite normal histology of the resting muscle and normal muscle stem cell behavior in the mutant mice, endothelial cells displayed an increase in the angiogenic response in resting muscle despite the downregulated transcriptomic changes induced by the CXCL12 mutation. The regenerative capacity of the CXCL12-mutated mice was only delayed after a notexin injury, but a severe damage by freeze injury revealed a persistent defect in the muscle regeneration of CXCL12 mutant mice associated with vascular defect and fibroadipose deposition with persistent immune cell infiltration.

**Conclusion:**

The present study shows that CXCL12 is crucial for proper muscle regeneration. We highlight that this homing molecule could play an important role in drastic muscle injuries and that the regeneration defect could be due to an impairment of angiogenesis, associated with a long-lasting fibro-adipogenic scar.

## Background

Wound healing is complex, and an alteration in the process can lead to chronic wound or fibrotic tissue formation. Skeletal muscle owns its remarkable capacity of regeneration to satellite cells (SCs), the main stem cells of this tissue [1]. After muscle injury, SCs are activated, proliferated, differentiated, and fused to repair damaged myofibers [2]. However, muscle regeneration also requires specific structural and trophic cues such as the presence of functional vascular supply and neuronal activity [3, 4].

The stem cell’s microenvironment is composed of a multitude of signals secreted by neighboring cells. Among chemokines, CXCL12 (SDF-1) is one of the most studied and was initially described to maintain hematopoietic stem cells within the bone marrow [5]. CXCL12 exists in three splice variants (α, β, and γ) in mice and acts on cells that are expressing the CXC chemokine receptor type 4 (CXCR4) and/or 7 (CXCR7) [6]. Beyond binding to their cognate receptors, the affinity of the chemokines to the glycan moiety of proteoglycans (GAG), specifically heparan sulfates (HS), creates a concentration gradient which drives an orientated migration and recruitment of circulating cells from surrounding tissues (diffusion/chemotaxis) [7].

CXCL12 has interesting proangiogenic properties of stimulating new blood vessel (vasculogenesis) formation during both the developmental and the postnatal period [8] and on angiogenesis [9]. Under ischemic conditions, CXCL12, upregulated by HIF1-α, leads to the mobilization of endothelial progenitor cells (EPCs) from the bone marrow [9] to revascularize injured tissues. Further, CXCL12 was described as a local factor in driving neovascular sprouting [10] and as having a direct effect on endothelial cells (EC) proliferation and in vitro capillary tube formation [11]. These beneficial effects have also been confirmed in vivo using a Matrigel plug assay [12].

Recent studies have also described the potential involvement of the CXCL12-CXCR4 pathway in the muscle repair process. An increase of CXCL12 was shown to enhance regeneration of injured skeletal muscles by inducing stem cell mobilization and by increasing the migration of myoblasts [13]. In addition, the CXCL12-CXCR4 pathway was shown to be upregulated in response to skeletal muscle damage and a CXCR4 antagonist induced a delay in muscle regeneration [13]. Thus, the administration of CXCL12 could accelerate the skeletal muscle repair process [13].

However, the involvement of chemokines, specifically the adsorption of CXCL12 by glycosaminoglycan and their biological functions, has not been elucidated. A new CXCL12 mutant mouse (CXCL12^Gagtm/Gagtm^ mice) was recently engineered, with the CXCL12 gene having been selectively mutated (knock-in) in the critical HS-binding domain supposed to induce drastic reduction of CXCL12/HS interaction without affecting CXCR4-activation capacity [14]. CXCL12^Gagtm/Gagtm^ (KI) mice showed no developmental defects and expressed normal levels of total and CXCL12 isoform-specific mRNA. The KI mice had an increase in the concentration of circulating CXCL12 and in circulating CD34+ hemopoietic precursor cells. However, after acute muscle ischemia, the KI mice exhibited a defect in revascularization [14].

We therefore investigated the role of the CXCL12/HS proteoglycan interactions in the pathophysiology of muscle regeneration, focusing on the SC behavior and in vascular abnormalities. When comparing severe muscle injuries which affected all cell types and disrupted the overall architecture of the muscle, the KI mice showed more fibroadipose scarring neighboring the regenerative regions relative to their wild-type counterparts. Thus, the disruption of the CXCL12/GAG interactions may transform the muscle into a non-regenerative tissue. An important aspect of this defect was the abnormal vascular system that developed in this fibro-adipogenic scar.

## Materials and methods

### Mouse experiments and muscle injuries

All procedures in this study were approved by the Animal Care and Use Committee at the Institut Pasteur (CETEA 01332.01). Unless specified, 6–10-week-old males were used in this study and housed under a 12:12 light/dark cycle in a pathogen-free facility with controlled temperature and humidity. Food and drink were given ad libitum*.* The experiments were conducted in C57BL/6 J RJ mice (Janvier Labs, France) or in genetically engineered mice backcrossed with making having a C57BL/6 J RJ background.

Animals were anesthetized by ketamine and xylazine (respectively 80 mg/kg and 10 mg/kg before injuries). For the freeze injury, the tibialis anterior (TA) was exposed and frozen with three consecutive cycles of freeze-thawings by applying a liquid nitrogen-cooled metallic rod for 15 s. For the myotexin injury, 10 μL of 12.5 μg/mL notexin (Latoxan) was injected in the TA. To limit the variability between the toxin batches, 25 batches (12.5 mg) were reconstituted, pooled, aliquoted, and stored at − 20 °C.

### Histological staining

TAs were collected and snap-frozen in liquid nitrogen-cooled isopentane for 5 min and stored at − 80 °C before cryosectioning (7 μm sections). The sections were then routinely stained with hematoxylin-eosin, Sirius Red, or Oil Red O.

To preserve GFP fluorescence for the 3D study of blood vessel organization, whole TA muscles were fixed in 10% neutral-buffered formalin for 2 h, then cryopreserved in 40% sucrose overnight at 4 °C before freezing in OCT (Tissue-Tek®, Sakura® Finetek, CA, USA). Serial cryosections (7-μm- or 100-μm-thick sections for 2D and 3D analysis, respectively) were performed.

For immunostaining, the tissues were rehydrated in PBS, saturated with 3% BSA, and permeabilized with 0.5% Triton X-100. Sections were incubated with primary antibodies (CD31, BD Pharmingen, #550274; Laminin, Sigma, #L9393; Pax7, DSHB) overnight at 4 °C and then with Alexa-conjugated secondary antibodies for 1 h at 37 °C. Sections were counterstained with Hoechst 33342 (Life technologies®, CA, USA) (5 min in PBS with 10 mg/mL, H3570, Invitrogen, CA, USA).

### Image acquisition

Images were captured on a Nikon Eclipse E800 microscope using the Nikon ACT-1 software and DXM1200 camera for bright field image acquisition. Fluorescence images and 3D reconstructions were performed with a Leica® TCS SPE DM 2500 and LAS AF software (Leica®, Germany).

Two-dimension analyses were performed, using ImageJ (NIH, MA, USA) and NIS-Element (Nikon) software. We measured the muscle fibers’ count, diameter, and capillary number per myofiber. At least 100 randomly selected fibers were considered for each muscle.

Three-dimensional analysis was performed to evaluate the organization of the vascular network and to quantify the number of vessels sprouting. For each muscle, 10 images were collected at 4 μm intervals to create a stack in the *z* axis. 3D reconstruction of this z-stack image was performed using 80- to 150-μm-thick frozen sections.

### Cell sorting and FACS

Using transgenic *Tg:Pax7nGFP* and Flk1^GFP/+^ mice allowed for selection by cytometry (FACS). Soft tissue was separated from the bone by dissection in cold DMEM, and muscles were chopped. Single cell suspensions were obtained from the TA or muscle bulk by enzymatic digestion at 37 °C with gentle agitation (collagenase (Sigma; T1426) 0.08% and trypsin (Sigma; C5138) 0.08% for SCs; collagenase B (Roche; 11088807001) 10 mg/mL and dispase II (Dutscher Dominique; 17105-041) 2.4 U/mL for other cell sorting).

After a 20-min digestion, the supernatant was collected on ice and enzymatic solution was added until the muscle was completely digested. The solution was filtered through 40 μm filter. To exclude dead cells, cells were stained by propidium iodide (Sigma, #P4170) prior to their analysis for the individual sorts of GFP-ECs and GFP-SCs. For cell sorting from uninjured and regenerating muscle of *Tg:Pax7nGFP*, ECs, macrophages, and fibro-adipogenic precursor cells were isolated using anti-FA/80-PE (Biolegend, #123110), anti-CD31-e450 (eBiosciences, #48-0311-82), anti-CD45-APC/Cy7 (Biolegend, #103116), and anti-Sca1-PE/Cy5 (Biolegend, #012463). Cell sorting was done with Aria III (BD Biosciences) and BD FACSDIVA software from BD Biosciences.

### Live video microscopy

Cells isolated by FACS were plated overnight on a 24-well glass bottom plate (P24G-0-10-F; MatTek) coated with Matrigel (BD Biosciences #354234) and placed in an incubator in pre-equilibrated medium (1:1 DMEM Glutamax:MCDB (Sigma-Aldrich), 20% fetal calf serum (FCS; Biowest S1860). The plates were then incubated at 37 °C, 5% CO_2_ (Zeiss, Pecon). A Zeiss Observer.Z1 connected with a LCI PlnN × 10/0.8 W phase II objective and AxioCam camera piloted with AxioVision was used. Cells were filmed for up to 5 days, and images were taken every 30 min with bright field and phase filters and MozaiX 3X3 (Zeiss).

### In vivo angiogenesis assay

To assess the residual capacity of endothelial cells to respond to a normal or modified CXCL12 signaling gradient, attractor cells (SCs), which have better angiogenic properties than others as previously described [15], from *Tg:Pax7nGFP* WT and KI mice were FACS isolated and culture expanded in 22 μm filtered 1:1 DMEM Glutamax (Gibco): MCDB (Sigma-Aldrich; M6770) containing 20% fetal bovine serum (FBS), plated on Matrigel (BD Biosciences; #354234). Cells were grown in an incubator (37 °C, 5%, CO_2_).

Cold Matrigel (BD Biosciences; #354234) was mixed with myoblasts (7.10^5^ cells/mL). Mice were anaesthetized using isoflurane, and cold Matrigel (0.5 mL) was injected into the abdominal subcutaneous tissue. After 21 days, mice were euthanized and Matrigel plugs were removed and fixed in JB fixative (zinc acetate 0.5%, zinc chloride 0.05%, and calcium acetate 0.05% in Tris buffer at pH = 7) for 48 h and then embedded in low-melting point paraffin (poly ethylene glycol distearate; Sigma, USA). Five-micrometer-thick paraffin sections were deparaffinized in absolute ethanol, air dried, and used for HE staining and immunolabeling for CD31/Desmin.

Blood vessel formation into the plug was quantified in one section per plug, by counting the number of cells per square millimeter using ImageJ software. Images were obtained on Leica® TCS SPE DM 2500 and LAS AF software (Leica®, Germany) microscope and expressed as mean values of 3–4 mice per condition.

### Vascular functionality imaging

Perfusion imaging was performed prior to and 28 days post-FI by dynamic contrast enhancement MRI (DCE-MRI). Briefly, a bolus of contrast agent, gadoterate meglumine, was injected intravenously and followed by T1-weighted MRI imaging for more than 9 min.

The data were analyzed using Tofts/Kety model, providing an estimated blood flow.

### Microarray analysis

RNA’s quality and concentration of satellite cells (GFP+) and endothelial cells (GFP+) respectively sorted by FACS from TA of *Tg:Pax7nGFP* and Flk1^GFP/+^ WT and KI mice were assessed with a 2100 Bioanalyzer (Agilent). RNA (RIN > 5) was processed and hybridized on a GeneChip™ Mouse Gene 2.0 ST Array (Affymetrix).

Raw data (.CEL files) were read into the R language and environment for statistical computing (version 3.4.1; R Foundation for Statistical Computing, Vienna, Austria; https://www.R-project.org/) using Rstudio (version 1.0.153; Rstudio, Boston, USA; https://www.rstudio.com/) and the affy package (version 1.54.0). Preprocessing and quality control was performed by using the oligo package version 1.40.2.

Array data were normalized using the Robust Multi-array Average (RMA) method and summarized by median polish.

Affymetrix probe identifiers were mapped to Entrez Gene identifiers using mogene20sttranscriptcluster.db package (version 8.6.0), and log-transformed intensities yielded by the RMA process were averaged per Entrez Gene IDd, producing 19.685 unique identifiers.

Corresponding Gene Ontology Biological Pathways (GOBP) were retrieved using gage package (version 2.26.1). In each cell type, for each GOBP, the null hypothesis was an absence of CXCL12 mutation effect, that is, a repartition of the between-group rank-test statistic of genes belonging to that GOBP following the same repartition that the overall gene between-group rank-test statistics.

Non-parametric rank tests were separately performed per cell type to test for a mutation effect, using the Generally Applicable Gene-Set/Pathway Analysis in gage package.

For a given GOBP, CXCL12 mutation effect was considered significant below a cutoff *q* value of 0.1 (*q* value resulting from the Benjamini-Hochberg procedure). The lists of significantly affected GOBP were simplified to GO-slim generic terms with GSEABase package (version 1.38.2).

### RTqPCR

Total RNA was isolated from cells using the RNAeasy Micro kit (Qiagen) and reverse transcribed using Superscript III Reverse transcriptase (Invitrogen). RTqPCR was performed using Power Sybr Green PCR Master Mix (Applied Biosystems), and the rate of dye incorporation was monitored using the StepOne Plus RealTime PCR system (Applied Biosystems). Two biological replicates were used for each condition. Data were analyzed by StepOne Plus RT PCR software v2.1 and Microsoft Excel. Rpl13 transcript levels were used for the normalization of each target (= ΔCT). RTqPCR CT values were analyzed using the 2^-(ΔΔCT)^ method. The primer sequences used are listed in Additional file 1: Table S1.

### Scanning electron microscopy

Samples were fixed at 37 °C in 0.05% glutaraldehyde and 2% PFA in 0.2 M Hepes for 15 min followed by fixation in 4% PFA in 0.2 M Hepes for 15 min. Samples were post-fixed in 2.5% glutaraldehyde in 0.2 M cacodylate buffer (pH 7.2) at 4 °C, washed three times for 5 min in 0.2 M cacodylate buffer (pH 7.2), treated for 1 h with 1% osmium tetroxide in 0.2 M cacodylate buffer, and then rinsed in distilled water. Samples were dehydrated through a graded series of 25, 50, 75, and 95% ethanol solutions for 5 min and for 10 min in 100% ethanol followed by critical point drying with CO_2_. Samples were sputtered with a 10-nm gold/palladium layer and were observed in a JEOL JSM-6700F field emission scanning electron microscope at a voltage of 5 kV. Pericytes and endothelial cells were identified as described by Sims [16].

### Statistical analysis

Unless specified, the data are expressed as mean ± SEM. When stated, the percentile 95% confidence intervals were calculated by bootstrapping the statistic using R (3.4.1) *boot* package. Statistical analysis was performed using GraphPad software (Prism, CA, USA) with the Mann-Whitney test or Wilcoxon signed rank test; *p* ≤ 0.05 was considered statistically significant.

Microarray data are presented in the form of volcano plots (integrating log2 fold values and multiple-test adjusted probabilities) and as heat map plots, generated in R studio.

## Results

### CXCL12 KI mice resting muscle has a less stabilized vasculature

**Fig. 1:**
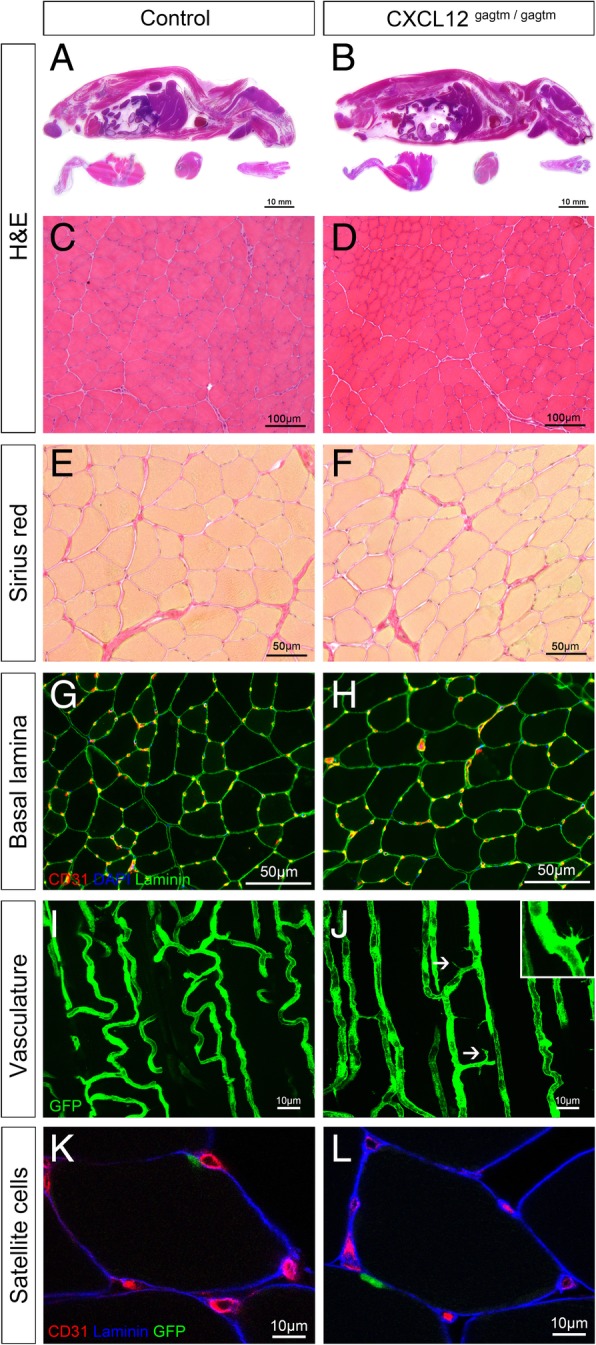
CXCL12^Gagtm/Gagtm^ mice show no systemic developmental or morphological defects but signs of vascular destabilization. **a**, **b** Representative pictures of HE-stained sagittal cross section of 8-week-old **a** WT (C57Bl6) mice and **b** KI (CXCL12^Gagtm/Gagtm^) mice. Scale bar represent 10 mm. Muscle morphology comparison between the KI and the WT mice on **c**, **d** HE stained tibialis anterior (TA) sections from **c** WT and **d** KI mice. Scale bar represent 100 μm. **e**, **f** Sirius Red (collagen deposits) staining in TA sections from **e** WT and **f** KI mice. Scale bar represent 50 μm. **g**, **h** Endothelium (CD31, red) and muscular basal lamina (laminin, green) immunolabeling counterstained for nuclei (DAPI, blue) in TA sections from **g** WT and **h** KI mice. Scale bar represent 50 μm. **i**, **j** Longitudinal blood vessel organization of sectioned TA from **i** WT (Flk1^GFP/+^) and **j** KI (CXCL12^Gagtm/Gagtm^:Flk1^GFP/+^) mice. White arrowheads point towards the sprouting structures. Scale bar represent 10 μm. **k**, **l** SC (Pax7-GFP, green) location, basal lamina (laminin, blue), and vessels (CD31, red) immunostained in TA sections from **k** WT (*Tg:Pax7nGFP*) and **l** KI (CXCL12^Gagtm/Gagtm^:Pax7nGFP) mice. Scale bar represent 10 μm (*n* = 3 animals per condition). All the experiments were repeated independently two times

To investigate the muscle morphology in the CXCL12^Gagtm/Gagtm^ knock-in (KI) mice [14], sagittal cross sections with hematoxylin-eosin (HE) staining of 8-week-old KI mice were performed. The stained cross sections did not reveal any malformation when comparing the KI mice to the C57Bl/6 mice (WT) (Fig. 1a, b). Specifically, extensive histological analyses on the skeletal muscle sections did not show any major differences between the WT and the KI mice. Tibialis anterior (TA) cross sections stained by HE showed muscle fascicles surrounded by a thin layer of perimysium individually separating each fiber with peripherally located nuclei (Fig. 1c, d). No differences were observed in either the number of the fibers nor the size of the fibers (Additional file 2: Figure S1A and S1B). In addition, the histological visualization of collagen I and III fibers by Sirius Red staining in the KI mice revealed typical organization of the connective tissues inclusive of the endomysium, the perimysium, and the epimysium layers (Fig. 1e, f; Additional file 2: Figure S1C). These data were confirmed by immunostaining against laminin, which is a component of the muscle basal lamina. Both the KI mice and the WT mice showed no abnormalities (Fig. 1g, h).

Next, to investigate the vasculature of the skeletal muscle, we assessed the number of capillary sections per myofiber (called myofiber “capillarization”) using laminin/CD31 immunolabeling (Fig. 1g, h). An unsignificant increase in the capillarization of the myofibers in the TA from the KI mice over the WT mice was observed (*p* = 0.09; Additional file 2: Figure S1D). Using Flk1^GFP/+^ (WT-Flk1) [17] and CXCL12^Gagtm/Gagtm^:Flk1^GFP/+^ (KI-Flk1) mice, which have green fluorescent endothelial cells (ECs), cytometric analysis also showed a trend to an increase in the number of muscle ECs in the KI-Flk1 mice when compared to the WT-Flk1 mice (*p* = 0.07; Additional file 2: Figure S1E). Moreover, 3D imaging analysis revealed a well-organized vascular network in both the KI-Flk1 and the WT-Flk1 mice (Fig. 1i, j). Interestingly, we observed the presence of tip cells only within the resting muscle of the KI mice (Fig. 1 j, inset), associated with a significant increase in the number of blood vessels sprouting compared to the muscle of WT mice (Additional file 2: Figure S1F).

To characterize SCs during homeostasis, we used *Tg:Pax7nGFP* (WT-Pax7) and CXCL12^Gagtm/Gagtm^:*Pax7nGFP* (KI-Pax7) mice in which the GFP reporter gene marks all of the SCs [18]. Histological analysis of the TA sections showed that the WT and the KI SCs were both located between the sarcolemma and the laminin-labeled basal lamina and were close to the CD31-labeled vessels (Fig. 1l, h; Additional file 2: Figure S1G). Additionally, both of the histological and the cytometric analyses showed that the numbers of the SCs in the TA were not different between the WT and the KI mice (Additional file 2: Figure S1H and S1I).

Since the organization of the connective tissues and the muscles fibers, including the SCs, was maintained, one can conclude that the disruption of the CXCL12/HS interactions did not result in major morphological modifications in the resting skeletal muscle. In contrast, the vascular structures in the KI mice displayed some abnormalities, which could reflect the destabilization of the vascular network by active angiogenesis due to sprouting.

### The unchallenged SCs retain their adaptive behavior despite transcriptomic changes induced by the CXCL12 mutation

**Fig. 2:**
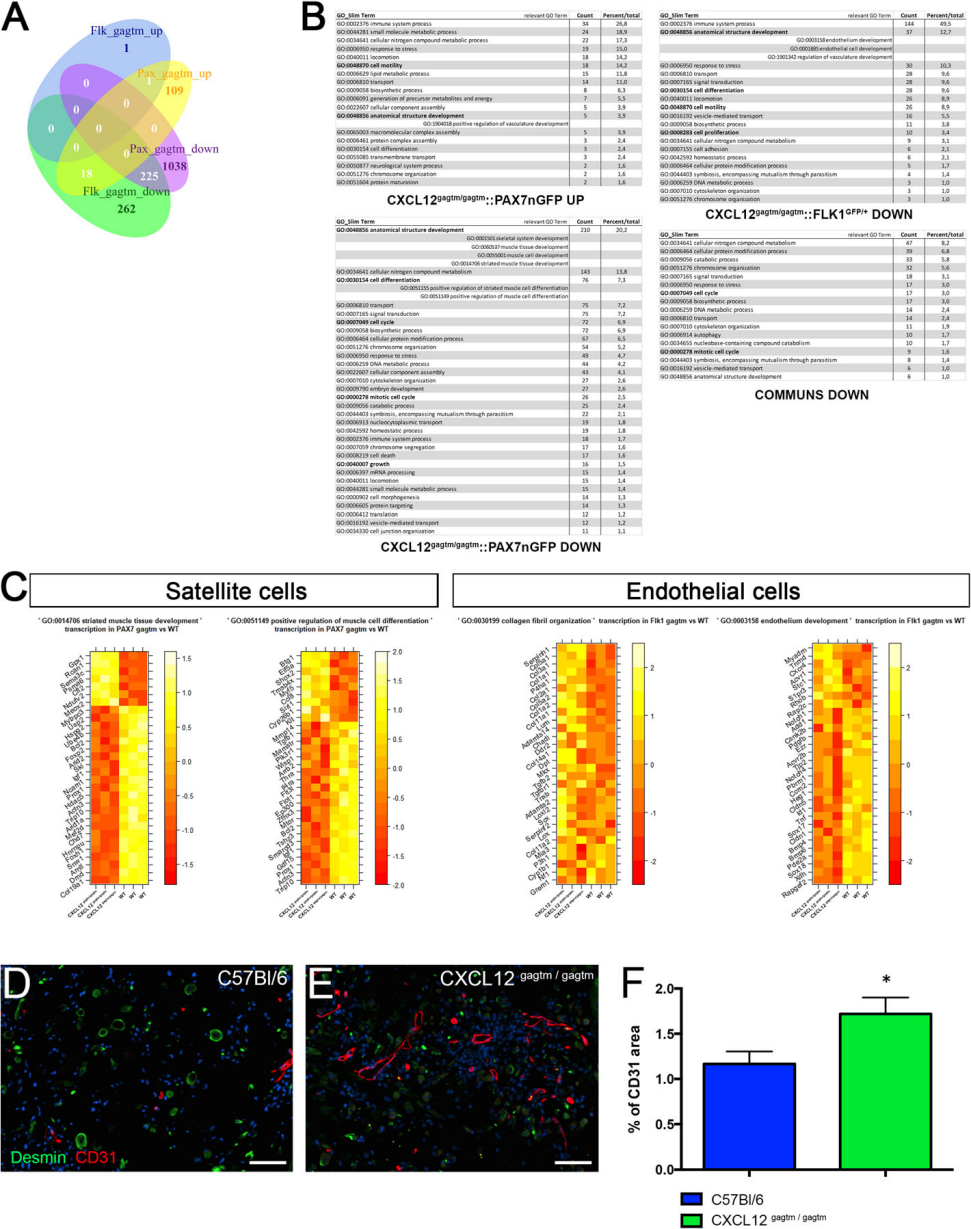
Transcriptome analysis of the SCs and the ECs from the uninjured TA of the KI versus the WT mice and in vivo angiogenesis assay. **a** Venn diagram of unique and overlapping differentially represented (*q* value < 0.1) Gene Ontology Biological Pathways (GOBP) in KI versus WT mice depending on the cell type (Pax7/GFP-positive and Flk1/GFP-positive cells). **b** Simplification of GOBP terms into GOBP slim terms, along with the number of differentially represented GOBP terms participating in each GOBP slim. **c** Transcriptional profile of the 30 most significantly differentially expressed genes in the selected GOBP. Expression of genes is presented as centered and scaled log2 fluorescence intensity (red to yellow key), and each row represents a gene, named by its MGI symbol (*n* = 3 animals were used per condition). **d**, **e** Representative immunostaining of Matrigel plugs mixed with KI (CXCL12^Gagtm/Gagtm^:Pax7nGFP) satellite cells inserted for 3 weeks in either **d** WT (C57Bl6) or **e** KI (CXCL12^Gagtm/Gagtm^) mice. Endothelial cells (CD31, red), myoblasts (desmin, green), and nuclei (DAPI, blue) were immunolabeling. Scale bars represent 20 μm. **f** Quantification of the CD31 positive/negative surface ratio as an indicator of vessel formation. Data are mean percentage ± SEM (10 fields per Matrigel plug). *n* = 5 animals per condition. All the experiments were repeated independently two times. *p < 0.05

To further investigate whether the CXCL12 mutation could affect the behavior of SCs at homeostasis, we performed a genome-wide microarray analysis on FACS-sorted SCs from the uninjured TA of WT-Pax7 and KI-Pax7 mice (Fig. 2a–c) confirmed by specific RTqPCR on the most up- and downregulated genes (Additional file 3: Figure S2A to S2C). The cell-type pathways involved in skeletal muscle development were specifically downregulated in the KI SCs, although no abnormalities were observed in the resting muscle (Fig. 1b, j). Strikingly, we showed that when compared to the WT mice, the cell cycle and cell differentiation-associated pathways were downregulated in the KI SCs. Conversely, the positive regulatory pathways of vasculature development including the CXCR4-related pathway were upregulated in the KI SCs.

To confirm whether the transcriptomic modifications induced by the CXCL12 mutation could modify the SCs’ behavior, we assessed, in vitro, the parameters of the FACS-sorted SCs. Video microscopy did not show any difference between the WT-Pax7 and the KI-Pax7 SCs with respect to the velocity (Additional file 3: Figure S2D), the onset of the first cell division (Additional file 3: Figure S2E), and the division rate (Additional file 3: Figure S2F). The percentage of MyoD+ (the SC activation marker) and Myogenin+ (the SC differentiation marker) cells explored at 2 days and at 4 days post-plating did not show any difference between the two types of SCs (Additional file 3: Figure S2G).

Overall, these results suggest that the CXCL12 mutation modifies, in vivo, the transcriptomic landscapes in SCs. However, the muscle development and the SC location are preserved, arguing for the existence of an effective coping mechanism during both embryogenesis and homeostasis. In addition, the intrinsic adaptive capacities of SCs including activation, proliferation, migration, or differentiation are maintained in vitro, suggesting that the CXCL12 mutation does not have a direct impact on the myogenic process.

### ECs display an increase in the angiogenic response in resting muscle despite the downregulated transcriptomic changes induced by the CXCL12 mutation

Because the resting muscle had an altered vascular network (Fig.1j), we investigated the impact of the CXCL12 mutation on the behavior of ECs during homeostasis. We therefore performed a genome-wide microarray analysis on FACS-sorted ECs from the uninjured TA of WT-Flk1 and KI-Flk1 mice confirmed by specific RTqPCR on the most up- and downregulated genes (Additional file 4: Figure S3A to S3C). Cell cycle and cell-differentiation-associated pathways were markedly downregulated in the KI ECs, as were the positive regulatory pathways for vasculature development (Fig. 2a–c). Surprisingly, when comparing the KI vs. the WT ECs, the organization and synthesis of collagen fibrils were the only upregulated pathway, with alpha1 chains of collagens I, II, III, and V being particularly upregulated (Fig. 2c).

To confirm the role of the CXCL12/HS disruption on EC behavior, we performed an in vivo angiogenesis assay using Matrigel plugs containing either KI-Pax7 or WT-Pax7 SCs grafted to either WT or KI recipient mice. In the Matrigel plugs containing the KI SCs grafted into the KI mice, the number of CD31-labeled vessels increased by 74.3% when compared to the WT mice (*p* = 0.0025; Fig. 2d–f). The newly formed vessels were functional as the lumen contained red blood cells but were strikingly misshaped in appearance (Additional file 4: Figure S3D). In addition, the percentage of CD31+ area was always higher in the Matrigel plugs containing the KI SCs grafted into the KI mice when compared to Matrigel plugs with the WT SCs grafted into either of the recipient mice (Additional file 4: Figure S3E). In contrast, we did not observe any difference in vessel formation whenever the WT SCs were used in the Matrigel plugs, in either the WT or the KI recipient mice (Additional file 4: Figure S3E).

Taken together, these results suggest that the CXCL12 mutation indirectly induced a downregulated transcriptomic landscape in ECs, although these cells exhibited an increased proangiogenic response. However, the genetic profile of the ECs indicated that the CXCL12 mutation may impact the regenerative response-associated gene expression in the KI ECs. To test this hypothesis, we studied the muscle regeneration in WT and KI mice.

### The regenerative capacity of the CXCL12-mutated mice is delayed after a notexin injury

**Fig. 3:**
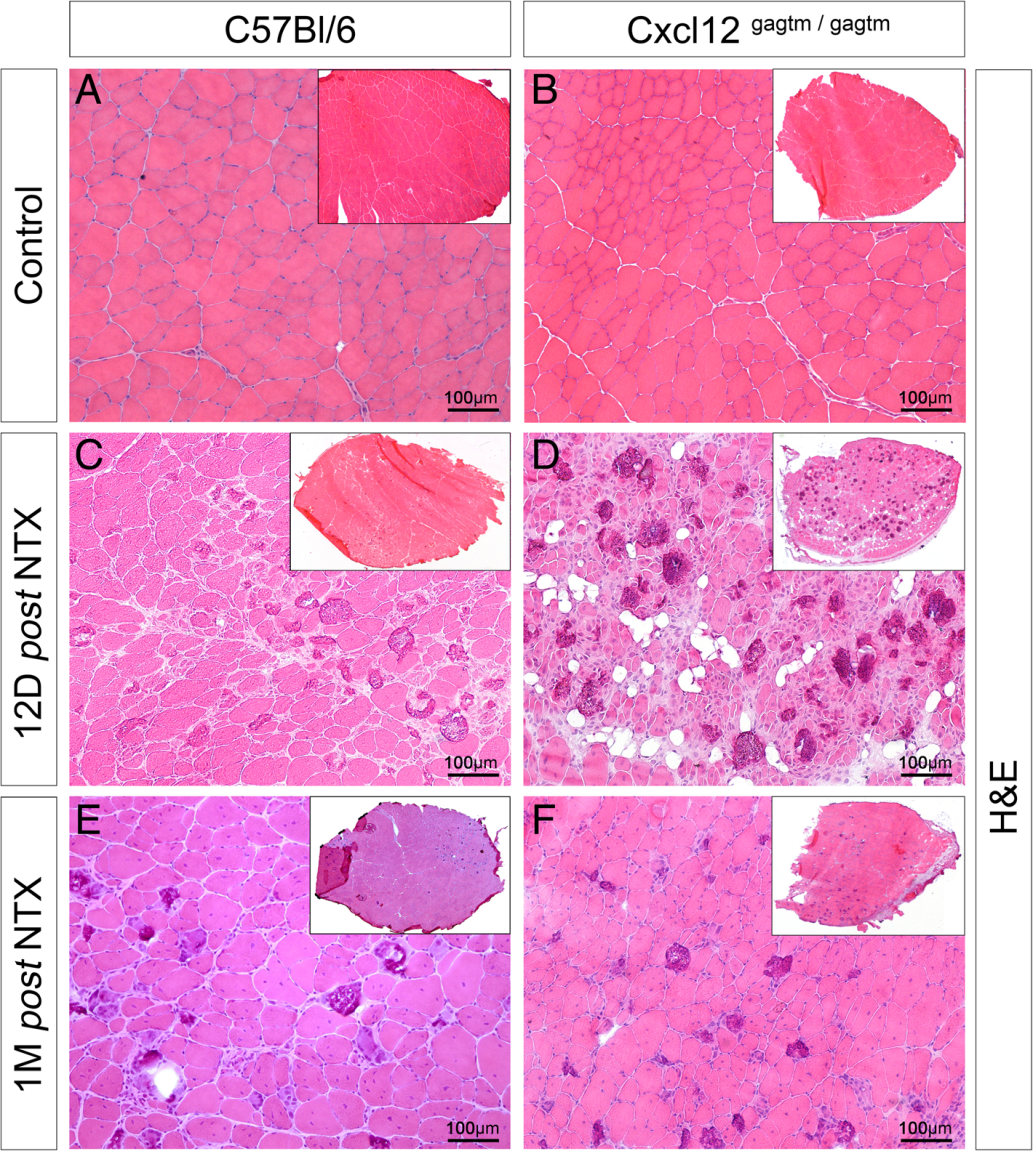
Muscle regeneration is delayed after a notexin injury in CXCL12^Gagtm/Gagtm^ mice. **a**–**f** Representative HE-stained TA sections respectively before 12 and 30 days post*-*NTX injury in **a**, **c**, **e** WT (C57Bl6) and **b**, **d**, **f** KI (CXCL12^Gagtm/Gagtm^) mice. Scale bar represent 100 μm. *n* = 3 animals per condition. All the experiments were repeated independently two 49 times

To investigate whether the CXCL12 mutation could have an effect during muscle regeneration, we began our investigation with a notexin (NTX) injection injury model that is less toxic for ECs and SCs [19]. At 12 days post-injury, the WT muscles showed partial regeneration. It was characterized by centrally nucleated small basophilic fibers that were associated with some peripheral multifocal calcium deposits, which replaced necrotic myofibers via a foreign body granulomatous reaction (Fig. 3c). Contrary to the WT muscles, the KI muscles displayed a delay in muscle regeneration with a significant increase in the number of calcium deposits (Additional file 5: Figure S4A and S4B) and with an unusual fat infiltration (Fig. 3d). One-month post-injury, the muscles from the WT and the KI mice were similarly regenerated with centro-localized nuclei (Fig. 3e, f) without any significant modification in the number of the fibers or in the size of the fibers (Additional file 5: Figure S4C and S4D). However, the KI muscles displayed a diminished increase in the myofiber’s capillarization when compared to the WT group (Additional file 5: Figure S4E). Cytometric analysis revealed that both of the KI-Pax7 and the WT-Pax7 mice showed an increase in the number of SCs in the injured TA 1 month post-NTX injury (Additional file 5: Figure S4F).

The CXCL12 mutant mice exhibited a delay of muscle regeneration 12 days post-NTX injury, followed by a complete regeneration after 1 month post-injury.

### Severe damage by freeze injury reveals a persistent defect in the muscle regeneration of CXCL12 mutant mice

**Fig. 4:**
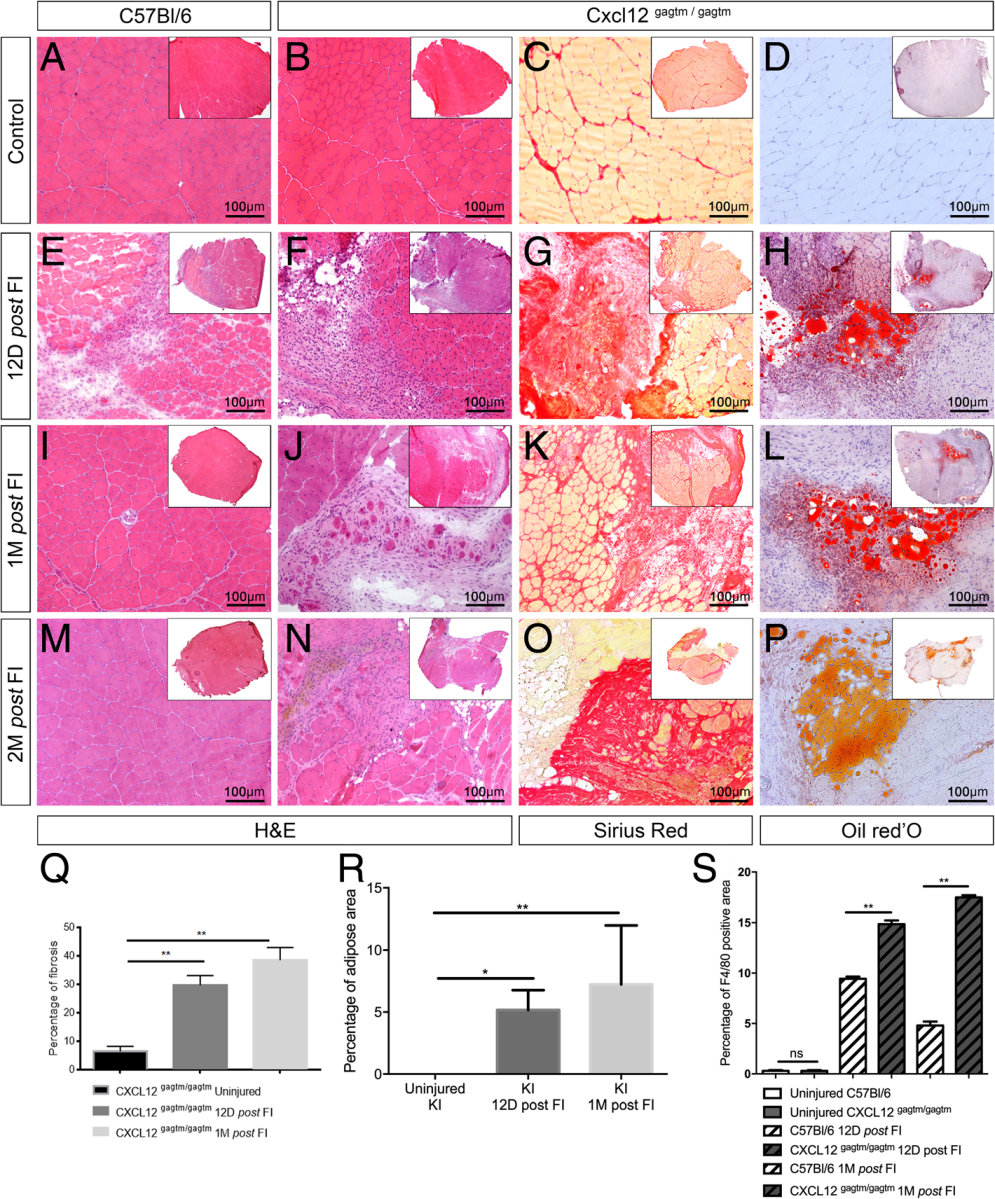
Freeze injury induces an impaired muscle regeneration with a long-lasting fibro-adipogenic scar in CXCL12^Gagtm/Gagtm^ mice. Representative HE-stained TA sections respectively before 12, 30, and 60 days post-FI in **a**, **e**, **i**, **m** WT (C57Bl6) and **b**, **f**, **j**, **n** KI (CXCL12^Gagtm/Gagtm^) mice. Scale bar represent 100 μm. Representative fibrotic deposit staining (Sirius Red) of **c** resting, **g** 12, **k** 30, and **o** 60 days post*-*FI TA from KI mice. Scale bar represent 100 μm. Representative adipocytic staining (Oil Red O) of **d** resting, **h** 12, **l** 30, and **p** 60 days post*-*FI of the TA from KI mice. Scale bar represent 100 μm. **q** Fibrosis quantified with Sirius Red positive/negative surface ratio. The mean ratio ± SEM is given for the injured TA from KI mice at three time points. **r** Adipose area quantified with Oil Red O positive/negative surface ratio. The mean ratio ± SEM is given for the injured TA from KI mice at three time points. **s** Macrophages infiltration quantified by F4/80 positive/negative surface ratio. The mean ratio ± SEM is given for injured TA from WT and KI mice at three time points. For all quantifications, *n* = 5 mice per condition and per time point and were repeated independently two times. *p < 0.05; **p < 0.01

Next, we used a freeze injury (FI) model which induced a greater destruction of fibers, SCs, and vessels in the muscles. Twelve days post-FI, the WT mice showed a regeneration wave composed of necrotic fibers located at the superficial layer, infiltration of inflammatory cells into the intermediate layer, and regenerated myofibers in the deep layer of the tissue (Fig. 4e). Interestingly, in WT mice, we showed that total CXCL12 gene expression was upregulated at 12 days post-FI (mean expression relative to reference gene 0.06 [CI_95_ 0.03–0.08] in uninjured, 1.90 [1.53–2.34] in FI mice, p = 0.03; Additional file 6: Figure S5A). Among the tested cell types, CXCL12 was specifically expressed by the fibro-adipogenic precursor cells (FAPs) and the ECs. In addition, the FAPs mainly expressed the isoform alpha of the CXCL12 gene in contrast to the ECs that expressed the gamma isoform (Additional file 6: Figure S5B to S5D). One and two months post-FI, the muscle was fully regenerated in the WT mice (Fig. 4i, m). This specific timing of the muscle regeneration was not observed in the KI mice. At 12 days post-FI, the KI muscles showed no area of regeneration (Fig. 4f), had a larger area of fibrosis in the Sirius Red staining (*p* = 0.0021; Fig. 4g, q), and displayed an unusual fat infiltration visualized by Oil Red O staining (*p* = 0.047; Fig. 4h, r). Moreover, whereas mutation was not significantly associated with a difference in CXCL12 gene expression in uninjured mice (mean difference KI–WT − 0.03 [CI_95_ − 0.06–0]; *p* = 0.27), this expression was significantly lower for KI mice post-FI as compared to their WT countertypes (mean difference KI–WT − 1.08 [CI_95_ − 1.57 to − 0.65]; *p* = 0.007; Additional file 6: Figure S5A to S5D). These regenerative defects lasted up to 2 months post-FI (Fig. 4j–l, n–p) with a progression of the fibrosis (*p* = 0.0011; Fig. 4q) and of the fat infiltration (*p* = 0.0079; Fig. 4r). The KI mice also exhibited an increase in macrophage (F4/80+ cells) infiltration 12 days post-FI when compared to the WT mice. This continued to increase in the KI mice 1 month post-FI, while they were transient in the WT mice (*p* = 0.0079; Fig. 4s). The muscle fibers were also affected in the KI mice at 1 month post-FI. Their size was reduced (Additional file 6: Figure S5F) and had a trend of reduced number of capillaries (Additional file 6: Figure S5G). In addition, we observed a drastic decrease (− 72 %) in the number of SCs (Additional file 6: Figure S5H) in the KI muscles when compared to the WT muscles.

These results suggest that after a severe damage, the muscle regeneration capacity is severely impaired by the CXCL12 mutation. Specifically, this pathological repair affects all actors involved in muscle regeneration, resulting in fibroadipose deposition and persistent immune cell infiltration, as well as a strong reduction in the number of SCs.

### The unstable vascular network is dysfunctional in the absence of the CXCL12/HS interaction during muscle regeneration

**Fig. 5:**
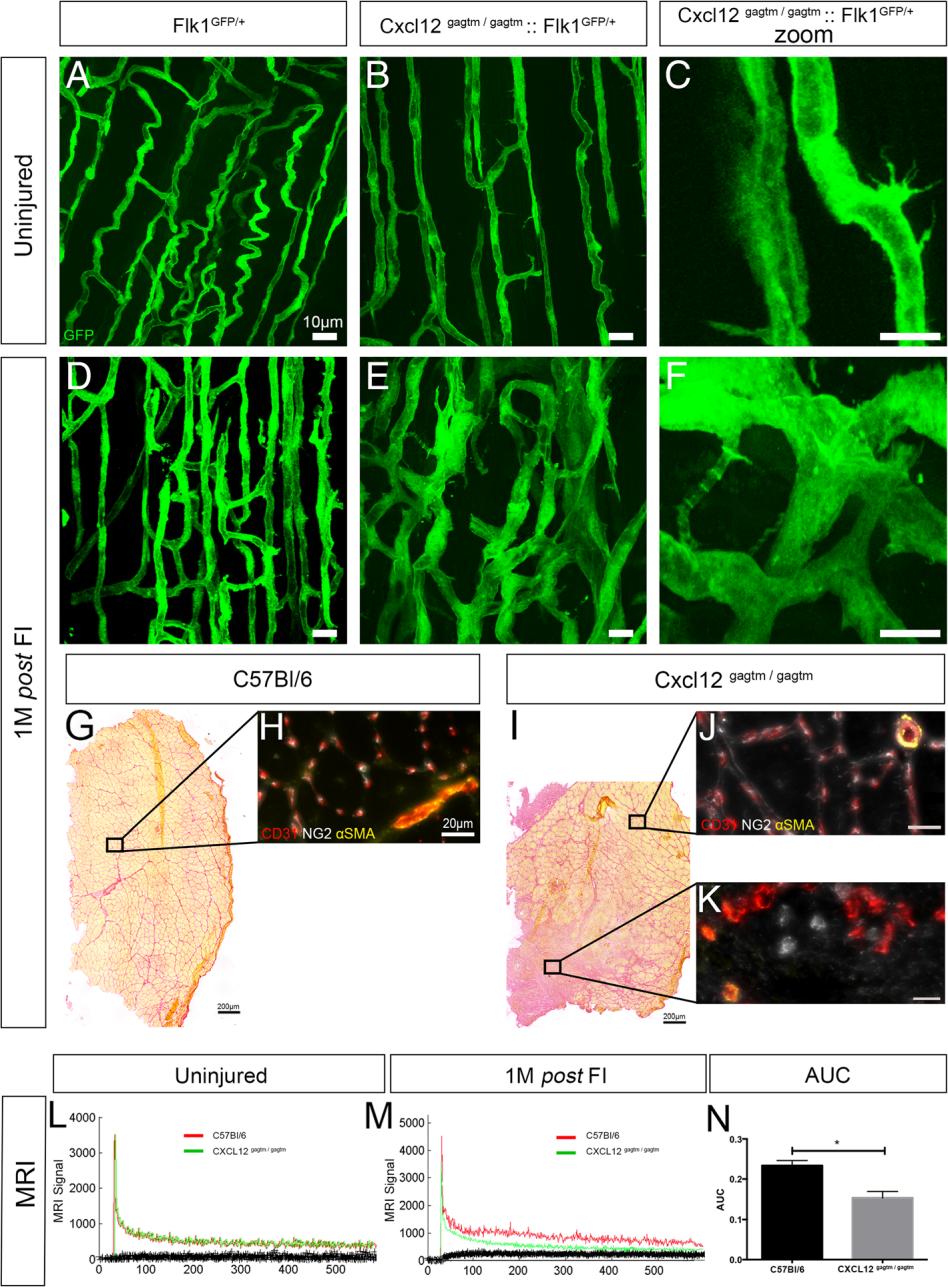
The abnormalities of vascular structures are associated with an absence of close contact with perivascular cells and with a functional perfusion defect post*-*FI in the KI mice. **a**–**f** Longitudinal blood vessel organization of the TA respectively before and 30 days post*-*FI in **a**, **d** WT (Flk1^GFP/+^) and **b**, **c**, **e**, **f** KI (CXCL12^Gagtm/Gagtm^:Flk1^GFP/+^) mice. Scale bar represent 10 μm. *n* = 3 animals per condition and per time point. All the experiments were repeated independently two times. Representative Sirius Red staining 1 month post*-*FI of the TA from **g** WT (C57Bl6) and **i** KI (CXCL12^Gagtm/Gagtm^) mice. Scale bar represent 200 μm. Immunostaining for ECs (CD31, red), pericytes (NG2, white), and smooth muscle cells (α-SMA, yellow) from the indicated zones of **h** injured TA from the WT, and **j** the regenerated or **k** the fibrotic TA from KI mice. Scale bar represent 20 μm. *n* = 3 animals per condition. All the experiments were repeated independently two times. **l**, **m** Direct contrast enhancement MRI assessment of vascular functionality: vascular tracer signal intensity per number of acquisitions comparing one WT (C57Bl6) to one KI (CXCL12^Gagtm/Gagtm^) mouse **l** before and **m** after 1 month post*-*FI. **n** The mean area under curve (AUC) ± SEM of vascular tracer intensity 1 month post*-*FI is given (*n* = 6 WT mice and *n* = 7 KI mice). *p < 0.05

To further study the impact of the CXCL12 mutation on the vascular network during muscle regeneration, we also performed a FI in WT-Flk1 and KI-Flk1 mice. One month post-FI, the KI-Flk1 mice exhibited vascular regeneration defects, including abnormal fusions forming large syncytia and atrophic vessels (Fig. 5a–f), associated with an increase in sprouting vessels number compared to the WT-Flk1 mice (*p* = 0.005; Additional file 7: Figure S6A).

To investigate the destabilization of the vascular network 1 month post-FI, we performed coupled immunostaining of ECs (CD31+) with perivascular cell labeling of pericytes (NG2+) and smooth muscle cells (α-SMA+). Regenerative zones in the KI mice showed no difference with those of the WT mice. We observed that each muscle fiber was surrounded mostly by capillary structures and rare arteriolar vessels associated with the expected pericytes and smooth muscle cells (Fig. 5g–j). Conversely, in the fibrotic regions observed only in the KI mice, large vascular structures were identified in the absence of close contact with perivascular cells (Fig. 5k). In addition, these vascular structures often appeared non-permeable, indicating a potentially functional defect (Fig. 5k).

To further confirm the abnormalities in vascular and perivascular structures post-FI, muscle samples were analyzed by scanning electron microscopy (SEM). In the WT muscles, we observed the specific localization of a SC. It was located between the basal lamina and the myofiber and was close to an EC which was covered by a pericyte (Additional file 2: Figures S6B to S6D). In the KI muscles, the two different zones of regeneration were selected. In the regenerated area, the muscle ultrastructure was similar to the WT muscle (Additional file 7: Figure S6F). In contrast, in the non-regenerated area, the number of pericytes identified around the vessels was significantly reduced compared to the WT muscle (*p* = 0.004; Additional file 2: Figures S6G and S6H). The EC was also isolated from the other structures by a large accumulation of fibrotic tissue (Additional file 7: Figure S6G).

To identify the functional impact of the CXCL12 mutation on the vascular network, muscle vascularization was examined by DCE-MRI comparing the WT and KI mice (Fig. 5l to n). Despite vascular abnormalities at resting condition, no significant difference was found in any functional parameters of the resting muscles of the KI and the WT mice (Fig. 5l). One-month post FI muscles from the KI mice exhibited a significant decrease in the perfusion parameters when compared to the WT mice (*p*=0.01; Fig. 5m and n). The blood flow between the injured and the contralateral muscle was lower in the KI group compared to the WT group (WT: 25.31 ± 16.8 mL/min/100mL KI: 17.02 ± 14.338 mL/min/100mL *p*=0.04) (data not shown).

These observations suggest that in the absence of the binding between CXCL12 and HS, the vascular network displayed local and heterogeneous destabilization during muscle regeneration. These abnormal vessels, having lost their cellular contact with the perivascular supporting cells, lead to a functional defect in muscle perfusion.

## Discussion

Our data indicates that high-affinity CXCL12 binding to HS plays a critical role in muscle regeneration following severe injury. However, the interaction does not appear to be critical in mild injuries nor in limb morphogenesis. We showed that disruption of the CXCL12/GAG interactions leads to a loss in the regenerative capacity which involves vascular abnormalities and the persistence of fibroadipose tissue remodeling.

HS synthesis has been shown to increase and to be critical in muscle regeneration in mice [20] and in axolotl models [21]. Among the GAG binding moieties, the CXCL12γ affinity for HS has been shown to be among the strongest ever reported for a protein [22]. This interaction has been proven to create concentration gradients crucial in the directional migration and the local retention of cells, as well as, enhancing the presentation of receptors and protecting ligands from degradation [23].

More specifically, the quantitative modulation of the CXCL12 signaling axis has already been shown to influence the kinetics and efficiency of muscle regeneration, and the volumetric muscle loss of age-associated cachexia [13, 24, 25]. CXCL12 has been shown to positively influence muscle repair by modulating SC migration [13, 24] and fusion [26] and by attracting non-muscle progenitor cells [24] with in vitro dose effects depending on the CXCL12 matrix adsorption [27]. To our knowledge, no studies have so far been conducted on the in vivo confirmation of the necessity of the CXCL12/HS binding during the muscle repair processes. Overall, the in vivo study on the CXCL12/GAG interactions which modulates a concertation gradient and/or the presentation of the molecule by the cell membrane during the skeletal muscle regeneration is a novelty.

As previously described, CXCL12^Gagtm/Gagtm^ mice reached the adult state and showed no morphological changes [14]. More specifically, the KI mice exhibited a normal histological organization of skeletal muscle. Although basal transcriptomic analysis of in vivo SCs revealed a dysregulation in major intracellular and developmental-related pathways, the SCs were normal in location and number. The SCs in vitro demonstrated the preserved abilities of migration, activation, proliferation, and early differentiation. Although this in vitro phenotype has not been studied in exogenous CXCL12-free conditions, it is indicative of a lack of cumulative alterations in the KI SCs during muscle damage.

Contrary to the appearance of a normal phenotype of the KI SCs, the KI mice exhibited minor vascular abnormalities in the non-challenged muscle, including an increase in the number of sprouting vessels. These could be linked with the presence of tip cells, which could be a marker of unstable vessels. However, this increased muscle capillarization did not translate into an enhancement in limb perfusion in the angio-MRI of the uninjured KI mice, thereby hinting at an inefficient vascularization. Interestingly, we showed in an in vivo Matrigel plug assay that the KI vessels exhibited increased angiogenic capacity only when the KI SCs where used as bait. These results were contrasting with the transcriptomic analysis of the ECs from the uninjured KI mice, which revealed the downregulation of pathways involved in proliferation, differentiation, and motility. Conversely, no increase in neoangiogenesis in response to the WT SCs grafted in the KI mice could be found. This is observed despite other cells from the KI mouse being free to colonize the Matrigel plug and secrete mutated CXCL12. Although the potent interaction between the SCs and the ECs during in vivo angiogenesis assay has already been shown [15, 28, 29], the specific activation and response of the KI vessels only to the KI SCs, which are capable of producing and releasing mutated CXCL12, as we showed, or other CXCL12-regulated factors, remains to be explored.

To study the importance of the CXCL12/HS interaction during muscle regeneration, we chose two injury models differing in severity and tissue layer location. We have previously demonstrated in WT mice that NTX injury induced a reduced destruction of SCs and vessels in muscle compared to FI [19]. Contrasting to the remarkable regenerative capacity of the WT mice, the KI mice exhibited an impaired regenerative phenotype which was dependent on the severity of the injury model. In particular, the NTX-induced injury 12 days post-injury showed a broader disruption in tissue and was associated with a temporary adipogenic response in the KI mice. However, there was a similar adaptive response in the number of SCs in the KI and the WT mice. A comparable delay in the post-NTX muscle regeneration has also been described after the inhibition of the CXCL12/CXCR4 signaling with a CXCR4 antagonist [13]. Interestingly, severely FI muscles from the KI mice generated large fibrotic zones. This alteration was associated with the lack of an increase in the number of SCs in the KI mice. In addition, fibrosis is an active process which differs from the volumetric loss that is to be expected if the lack of efficiency of the regenerative myogenesis steps was the only process involved in the regeneration. We accordingly broadened the scope of our study to include cell types associated with fibrosis.

One striking observation in post-FI fibrotic zones was the infiltration of macrophages into the damaged areas. This last observation apparently contradicts published data in which systemic administration of non-HS binding CXCL12 was shown to inhibit haptotactic leukocyte migration to sites of inflammation [30]. This discrepancy could result from design differences between the studies. In the reported study, the effect of single dose, after 24 h, of a mutated CXCL12 introduced intravenously into WT mice was studied. Conversely, in our studies, the persistent expression of a mutated chemokine and the assessment of macrophage infiltration 12 and 30 days post-injury were analyzed. The difference in the mutation of CXCL12 involved in both studies could also be an explanation. The mutated CXCL12 expressed by the KI mice in our studies has been shown to retain agonist potency without desensitizing CXCR4 [23], which was not the case in the other study.

The most salient feature of the pathological phenotype post-FI was the uncoordinated infiltration of abnormal vascular structures. Despite the apparently angiogenic-facilitating phenotype of the KI ECs, their adaptation to the local needs proved to be defective in the KI mice, as the grossly misshapen capillaries found 1 month post-FI associated with a decrease in perfusion efficiency in angio-MRIs. As previously reported when CXCL12 is overexpressed in models of limb ischemia, it supports neoangiogenesis by attracting EPCs and by increasing the number of newly formed vessels leading to an increase in blood flow [9]. In addition, CXCL12 also participates in vascular remodeling by recruiting inflammatory cells and by directly attracting smooth muscle cells [31, 32]. More specifically, the disruption of the CXCL12/GAG interaction also leads to a defect in angiogenesis and neovascularisation in a limb ischemia model, which can be rescued by the administration of exogenous CXCL12γ [14].

Surprisingly, the transcriptomic assay of the uninjured ECs from the KI mice suggested the possibility that they might be heavily primed towards extracellular matrix synthesis, a feature that only appears after severe injury. This phenotype could be explained by a dysregulation of the endothelial pericyte and the endothelial smooth muscle cell interactions. Indeed, pericytes participate in vascular regulation and stabilization [33], but are also regulated by the endothelium. Pericytes have been shown to be one of the major sources of collagen-producing cells in a kidney fibrosis model [34]. The alteration of their interactions has been established in both the capillary rarefaction and in fibrosis induction [35]. Interestingly, it has been shown that the increased levels of CXCL12α and the low HS binding isoform of CXCL12 participate in the kidney and myocardial fibrosis. It has been shown to directly induce a collagen-synthetizing phenotype on perivascular cells, a phenotype amenable by CXCR4 antagonist therapy [36]. Additionally, the direct stimulating role of CXCL12 in the expression of matrix proteases could be hindered and the fibrosis could result not only from an increase in collagen expression, but also from its non-degradation [13, 24].

Finally, it has been shown that chemokines cooperate by directly modifying the availability and gradient formation of each other through the competition for GAG adhesion. The exclusion of CXCL12 from this competition could lead the other chemokines to be perceived more strongly by their receptors revealing non-CXCL12 effects [37].

## Conclusion

The present study shows that CXCL12 is crucial for proper muscle regeneration. We highlight that this homing molecule could play an important role in drastic muscle injuries and that the regeneration defect could be due to an inefficient angiogenesis. Endothelial cells’ transcriptional priming towards extracellular matrix synthesis in uninjured KI mice could represent another facet of vascular involvement in fibrosis, if further experiments were to confirm the actual extracellular matrix molecule expression. Whether comparable skews towards fibrosis are observed in otherwise regenerating models such as hepatectomy following CXCL12/HS interaction disruption should also warrant further studies. Finally, understanding whether unbound CXCL12 alone triggers a profibrotic activity, HS-bound CXCL12 an antifibrotic activity, or whether an indirect mechanism is at play such as the displacement from HS disrupting the pattern of bound molecules could offer new insight in the occurrence of pathological scarring.

## Supplementary information

**Additional file 1: Table S1.** Oligonucleotide primers for RTqPCR, Related to Figures 5, **S2**, **S3**.

**Additional file 2: Figure S1.** (Related to Figure 1). Quantitative histological and cytometric parameters of the resting muscle from WT and CXCL12Gagtm/Gagtm mice. Quantification of (A) fibers number and (B) fibers diameter by Hematoxylin-eosin staining in the uninjured TA from WT (C57Bl6) and CXCL12Gagtm/Gagtm mice. Three animals (n=3) were used per condition and were repeated independently two times. (C) Quantification of Sirius Red positive/negative surface ratio. The mean ratio ± SEM (3 sections per mice, 10 images per sections) is given for the uninjured TA from KI (CXCL12Gagtm/Gagtm) mice. (D) Quantification of vessels number by CD31 immunostaining in the uninjured TA from WT (C57Bl6) and CXCL12Gagtm/Gagtm mice. Three animals (n=3) were used per condition and were repeated independently two times. (E) Quantification of GFP-positive cells by FACS analysis from both the TAs of WT (*Flk1GFP/+*) vs. KI (CXCL12Gagtm/Gagtm :: *Flk1GFP/+*) mice. (n=5 mice per condition). (F) Quantification of the sprouting vessels number in the resting muscle of WT (*Flk1GFP/+*) and KI (CXCL12Gagtm/Gagtm :: *Flk1GFP/+*) mice (n=5). Quantification of number of SCs by Pax7/GFP immunostaining (G) or by FACS analysis (H) in the uninjured TA from WT (*Tg:Pax7nGFP*) and CXCL12Gagtm/Gagtm mice (n=5 mice per condition). Data are given as the mean ± SEM. **** p < 0.0001.

**Additional file 3: Figure S2.** (Related to Figure 2). CXCL12gagtm/gagtm and WT satellite cells show identical behavior. (A) Heat map of the three most up- and down-regulated genes identified by the genome-wide microarray analysis on FACS-sorted SCs from the uninjured TA of WT-Pax7 (n=3) and KI-Pax7 (n=3) mice. Expression of genes is presented as centered and scaled log2 fluorescence intensity (red to yellow key), each row represents a gene, named by its MGI symbol. Confirmation by specific RTqPCRs for the three most up-regulated genes (B) and the three most down-regulated genes (C). Data are represented as the fold change of expression in KI SCs (n=5) compared to WT SCs (n=5) with the use of Wilcoxon signed rank test. SCs from (n=3) WT (*Tg:Pax7nGFP*) and (n=3) KI (CXCL12Gagtm/Gagtm::Pax7nGFP) were sorted by FACS and plated to assess their behavior by live videomicroscopy: (D) the velocity, (E) the onset first cell division and (F) the division rate. (n=100 cells counted). (G) Quantification by immunostaining 2 days *post* plating of percentage of MyoD and Pax7 population in WT vs KI SCs and 4 days *post* plating of percentage of Myogenin and Pax7 population in WT vs KI SCs (n=3 mice per condition and per time point). Data are given as the mean ± SEM. * p < 0.05; ** p < 0.01.

**Additional file 4: Figure S3.** (Related to Figure 2). KI ECs display an increased angiogenic response. (A) Heat map of the three most up- and down-regulated genes identified by the genome-wide microarray analysis on FACS-sorted ECs from the uninjured TA of WT-Flk1 (n=3) and KI-Flk1 (n=3) mice. Expression of genes is presented as centered and scaled log2 fluorescence intensity (red to yellow key), each row represents a gene, named by its MGI symbol. Confirmation by specific RTqPCRs for the three most up-regulated genes (B) and the three most down-regulated genes (C). Data are represented as the fold change of expression in KI ECs (n=5) compared to WT ECs (n=5) with the use of Wilcoxon signed rank test. (D) Representative immunostaining of matrigel plugs mixed with KI (CXCL12Gagtm/Gagtm::Pax7nGFP) satellite cells inserted for 3 weeks in KI (CXCL12Gagtm/Gagtm) mice with endothelial cells (CD31, red) and myoblasts (Desmin, green). Scale bars represent 10 μm. (E) Quantification of the CD31 positive/negative surface ratio in matrigel plugs mixed with the KI (CXCL12Gagtm/Gagtm::Pax7nGFP) or the WT (*Tg:Pax7nGFP*) SCs inserted for 3 weeks in either the WT (C57Bl6) or the KI (CXCL12Gagtm/Gagtm) mice. Data are mean percentage ± SEM (10 fields per matrigel plug). Five animals (n=5) were used per condition and were repeated independently two times. Data are given as the mean ± SEM.* p < 0.05; ** p < 0.01.

**Additional file 5: Figure S4.** (Related to Figure 3). Quantitative histological and cytometric parameters of the muscle one month *post* NTX injury in the WT and the CXCL12Gagtm/Gagtm mice. (A) Quantification of calcium deposits number by Von Kossa staining in 12 days and one month *post* NTX injured TA from WT (C57Bl6) and CXCL12Gagtm/Gagtm mice. Three animals (n=3) were used per condition and were repeated independently two times. (B) Representative Von Kossa stained TA section 12 *post* NTX injury in KI (CXCL12Gagtm/Gagtm) mice. Scale bar represent 100μm. Quantification of (C) fibers number and (D) fibers diameter by Hematoxylin-eosin staining in uninjured and *post* NTX injured TA from WT (C57Bl6) and CXCL12Gagtm/Gagtm mice. Three animals (n=3) were used per condition and were repeated independently two times. (E) Quantification of vessels number by CD31 immunostaining in the uninjured and the *post* NTX injured TA from WT (C57Bl6) and CXCL12Gagtm/Gagtm mice. Three animals (n=3) were used per condition and were repeated independently two times. (F) Quantification of GFP-positive cells by FACS analysis per TA of the uninjured and the *post* NTX injured WT (*Flk1GFP/+*) vs. KI (CXCL12Gagtm/Gagtm :: *Flk1GFP/+*) mice. (n=5 mice per condition). Data are given as the mean ± SEM. * p < 0.05; ** p < 0.01, *** p < 0.001.

**Additional file 6: Figure S5.** (Related to Figure 4). Quantitative histological and cytometric parameters of the muscle one month *post* FI in WT and CXCL12Gagtm/Gagtm mice. RTqPCR gene expression in FACS-sorted MPs, FAPs, SCs, ECs from TA muscle without injury and TA 12 days *post* FI from WT-Pax7 and KI-Pax7 with relative expression, represented as a log2, of total CXCL12 gene (A), alpha CXCL12 isoform gene (B), beta CXCL12 isoform gene (C) and gamma CXCL12 isoform gene (D). The data are represented in Log2 and n=3 animals per condition for uninjured mice, n=5 animals for injured WT-Pax7 mice and n=6 animals for injured KI-Pax7 mice. Quantification of (E) the number of fibers and (F) the fiber’s diameter by Hematoxylin-eosin staining in the uninjured and *post* FI TA from WT (C57Bl6) and CXCL12Gagtm/Gagtm mice. Three animals (n=3) were used per condition and were repeated independently two times. (G) Quantification of the number of vessels by CD31 immunostaining in the uninjured and the *post* FI injured TA from WT (C57Bl6) and CXCL12Gagtm/Gagtm mice. Three animals (n=3) were used per condition and were repeated independently two times. (H) Quantification of GFP-positive cells by FACS analysis per the TA of uninjured and *post* FI injured WT (*Flk1GFP/+*) vs. KI (CXCL12Gagtm/Gagtm :: *Flk1GFP/+*) mice. (n=5 mice per condition). Data are given as the mean ± SEM. * p < 0.05; ** p < 0.01, *** p < 0.001.

**Additional file 7: Figure S6.** (Related to Figure 5). Quantitative histological and ultrastructure parameters of the vascular network of muscle one month *post* FI in WT and KI mice. (A) Quantification of the sprouting vessels number one month *post* FI in the muscle of WT (*Flk1GFP/+*) and KI (CXCL12Gagtm/Gagtm :: *Flk1GFP/+*) mice (n=5). (B and E) Scanning Electron Microscopy (SEM) sample were selected according to Sirius Red staining. Scale bar represent 200 μm. (C and D) A set of representative images of a satellite cell (SC); a myofiber (MF); an endothelial cell (EC); a pericyte (PC) from WT (C57Bl6) muscle. Scale bar represent 1 μm. (F-G) Representative images of a SC, a MF a EC and a PC from two different zones selected on Sirius Red section in KI (CXCL12Gagtm/Gagtm) muscles: (F) in a fully regenerated area and (G) in the non-regenerated zone. Scale bar represent 1 μm. (H) Number of pericytes identified around vessels in the non-regenerated zone of KI muscle compared to WT muscle one month post FI. Ten fileds including at least one vessel were randomly counted on each TA section for each condition (n=3 animals per condition). Data are given as the mean ± SEM. p < 0.05; ** p < 0.01.

## Data Availability

The datasets used and/or analyzed during the current study are available from the corresponding author on reasonable request.

## Abbreviations

CXCR4: CXC chemokine receptor type 4
CXCR7: CXC chemokine receptor type 7
DCE-MRI: Dynamic contrast enhancement magnetic resonance imaging
EC: Endothelial cells
EPCs: Endothelial progenitor cells
FAPs: Fibro-adipogenic precursor cells
FBS: Fetal bovine serum
FI: Freeze injury
GAG: Glycan moiety of proteoglycans
GOBP: Gene Ontology Biological Pathways
HE: Hematoxylin eosin
HS: Heparan sulfates
KI: Knock-in
MPs: Macrophage
NTX: Notexin
SCs: Satellite cells
SEM: Scanning electron microscopy
TA: Tibialis anterior
WT: Wild-type
