# Supplementary material for: Defective angiogenesis in CXCL12 mutant mice impairs skeletal muscle regeneration
Source: Skelet Muscle. 2019 Sep 18;9:25. doi: 10.1186/s13395-019-0210-5 (PMC6751827; doi:10.1186/s13395-019-0210-5)
Supplement: Supplementary file 1 — Additional file 1: Table S1. Oligonucleotide primers for RTqPCR, Related to Figures 5, S2, S3. [file 13395_2019_210_MOESM1_ESM.docx]

**Table S1. Oligonucleotide primers for RTqPCR, Related to Figures 5, S2, S3.**

| Murine Primer | Forward sequence | Reverse sequence |
| --- | --- | --- |
| Rpl13 | 5’-CTCTGGCCTTTTCCTTTTTG-3’ | 5’-CCGAAGAAGGGAGAGTTC-3’ |
| Cry2 | 5’-CACTGGTTCCGCAAAGGACTA-3’ | 5’-CCACGGGTCGAGGATGTAGA-3’ |
| Nr1d1 | 5’-TACATTGGCTCTAGTGGCTCC-3’ | 5’-CAGTAGGTGATGGTGGGAAGTA-3’ |
| Mup20 | 5’-GTGCTGCTGCTGTGTTTGGG-3’ | 5’-TGTCAGTGGCCAGCATAATAGTA-3’ |
| Postn | 5’-CACGGCATGGTTATTCCTTCA-3’ | 5’-TCAGGACACGGTCAATGACAT-3’ |
| Laptm4b | 5’-CTCTGAACTAGGAGGGGAGTTT-3’ | 5’-GTTGCTTGTATGCACCGTAAGTA-3’ |
| Col1a1 | 5’-GCTCCTCTTAGGGGCCACT-3’ | 5’-CCACGTCTCACCATTGGGG-3’ |
| Upf3B | 5’-AGGAGAAACGAGTGACCCTGT-3’ | 5’-CCTGTTGCGATCCTGCCTA-3’ |
| Akap9 | 5’-ACAAGCACACACTCTATGTTCTC-3’ | 5’-TCGCCTAACTCTTTGCTAAGC-3’ |
| Bod1l | 5’-GCAAAGGCACGGGAAAATGG-3’ | 5’-TCGTCCGCCTCAATACTGGT-3’ |
| Cd36 | 5’-ATGGGCTGTGATCGGAACTG-3’ | 5’-GTCTTCCCAATAAGCATGTCTCC-3’ |
| Ly6a | 5’-AGGAGGCAGCAGTTATTGTGG-3’ | 5’-CGTTGACCTTAGTACCCAGGA-3’ |
| Lum | 5’-CTCTTGCCTTGGCATTAGTCG-3’ | 5’-GGGGGCAGTTACATTCTGGTG-3’ |
| Total CXCL12 | 5’-GCTCTGCATCAGTGACGGTA-3’ | 5’-TAATTTCGGGTCAATGCACA-3’ |
| CXCL12 alpha | 5’-TGCCCTTCAGATTGTTGCAC-3’ | 5’-CCACGGATGTCAGCCTTCC-3’ |
| CXCL12 beta | 5’-TGCCCTTCAGATTGTTGCAC-3’ | 5’-CTTGAGCCTCTTGTTTAAAGCTT-3’ |
| CXCL12 gamma | 5’-GCTAGCTTACAAAGCG  CCAGAGCAGAGCGCACTGCG-3’ | 5’-TGCCCTTCAGATTGTTGCAC-3’ |
