# Supplementary figures and images for: Defective angiogenesis in CXCL12 mutant mice impairs skeletal muscle regeneration
Source: Skelet Muscle. 2019 Sep 18;9:25. doi: 10.1186/s13395-019-0210-5 (PMC6751827; doi:10.1186/s13395-019-0210-5)

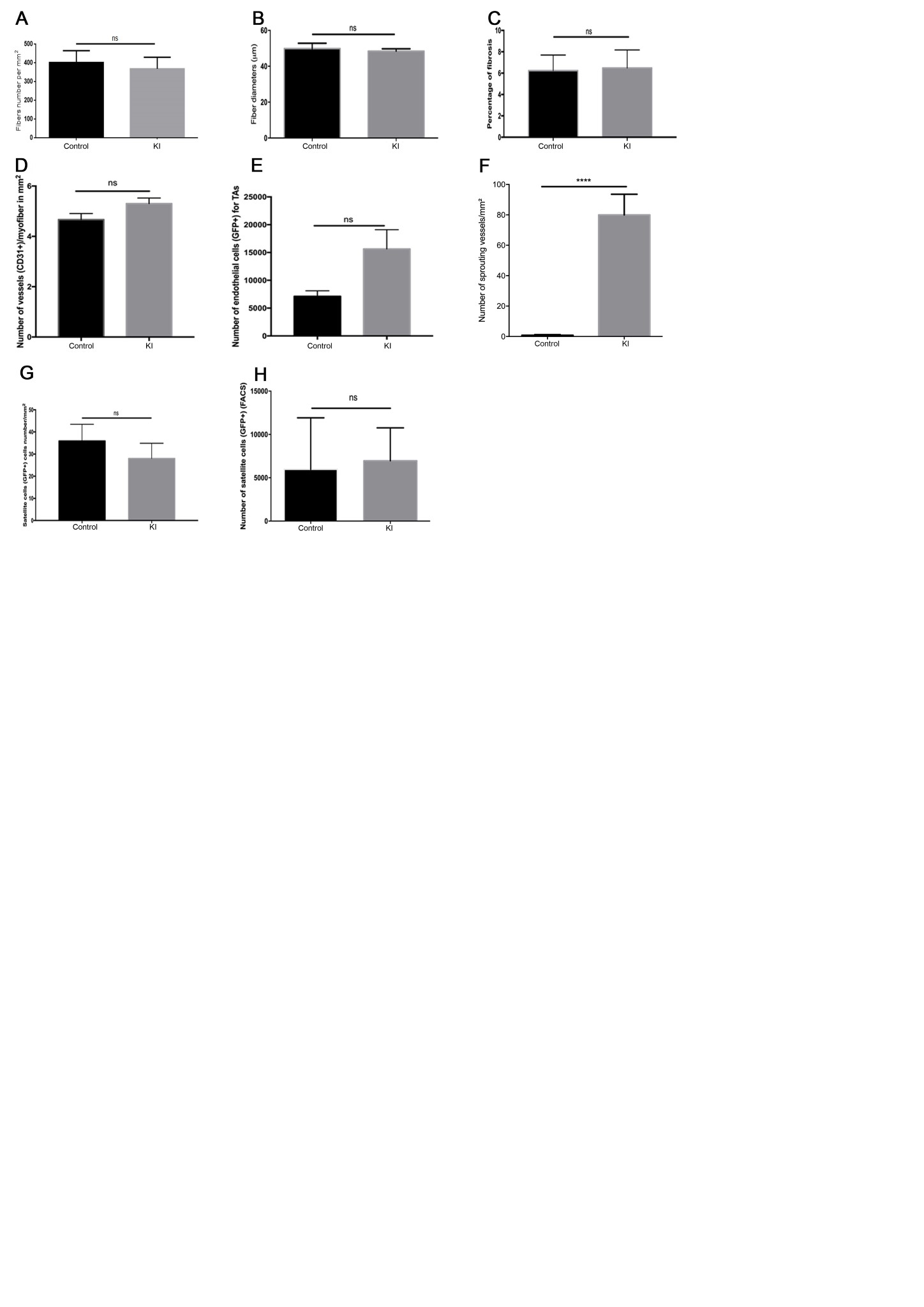

Supplement: Supplementary file 2 — Additional file 2: Figure S1. (Related to Figure 1). Quantitative histological and cytometric parameters of the resting muscle from WT and CXCL12Gagtm/Gagtm mice. Quantification of (A) fibers number and (B) fibers diameter by Hematoxylin-eosin staining in the uninjured TA from WT (C57Bl6) and CXCL12Gagtm/Gagtm mice. Three animals (n=3) were used per condition and were repeated independently two times. (C) Quantification of Sirius Red positive/negative surface ratio. The mean ratio ± SEM (3 sections per mice, 10 images per sections) is given for the uninjured TA from KI (CXCL12Gagtm/Gagtm) mice. (D) Quantification of vessels number by CD31 immunostaining in the uninjured TA from WT (C57Bl6) and CXCL12Gagtm/Gagtm mice. Three animals (n=3) were used per condition and were repeated independently two times. (E) Quantification of GFP-positive cells by FACS analysis from both the TAs of WT (Flk1GFP/+) vs. KI (CXCL12Gagtm/Gagtm :: Flk1GFP/+) mice. (n=5 mice per condition). (F) Quantification of the sprouting vessels number in the resting muscle of WT (Flk1GFP/+) and KI (CXCL12Gagtm/Gagtm :: Flk1GFP/+) mice (n=5). Quantification of number of SCs by Pax7/GFP immunostaining (G) or by FACS analysis (H) in the uninjured TA from WT (Tg:Pax7nGFP) and CXCL12Gagtm/Gagtm mice (n=5 mice per condition). Data are given as the mean ± SEM. **** p < 0.0001. [file 13395_2019_210_MOESM2_ESM.docx]

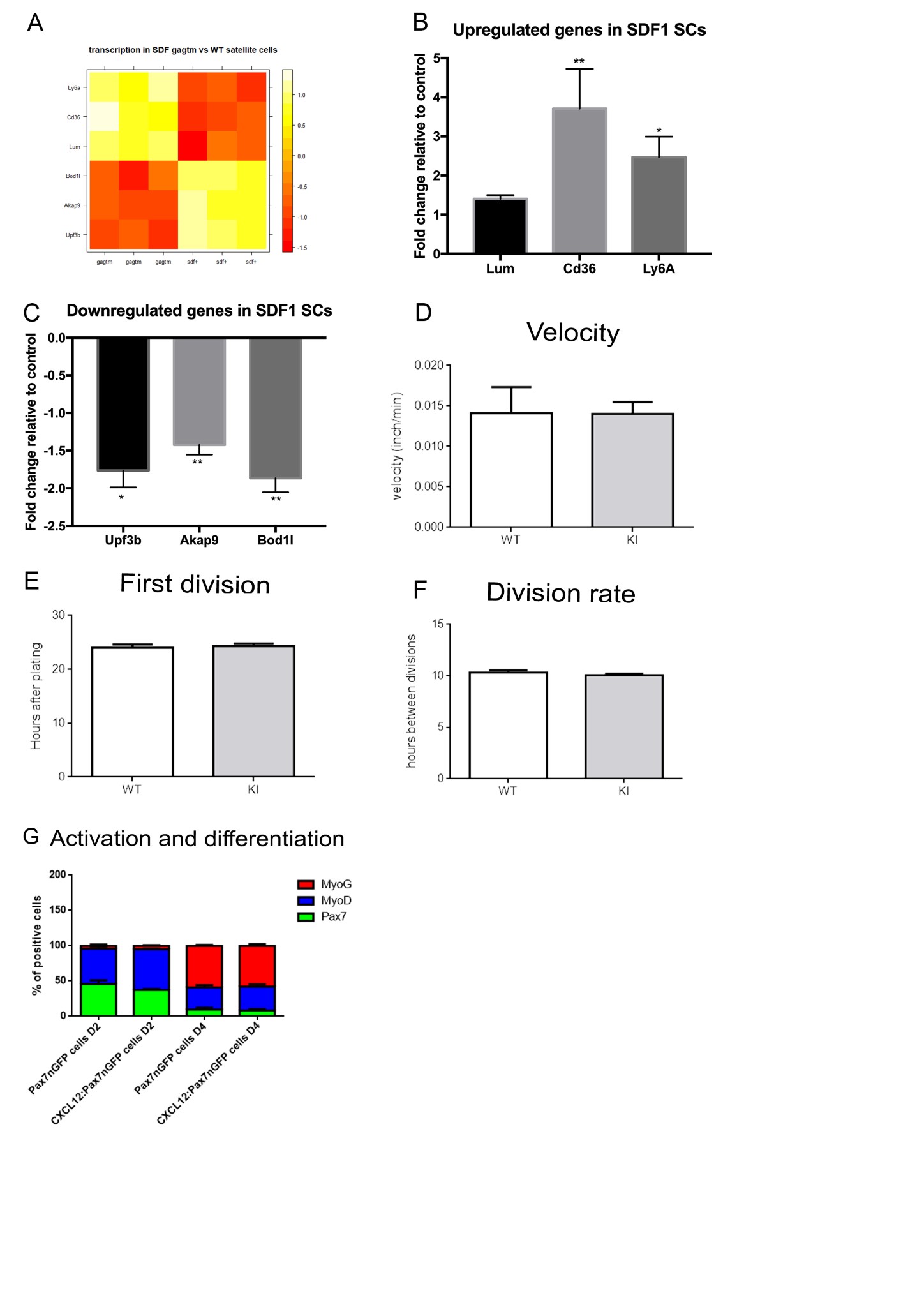

Supplement: Supplementary file 3 — Additional file 3: Figure S2. (Related to Figure 2). CXCL12gagtm/gagtm and WT satellite cells show identical behavior. (A) Heat map of the three most up- and down-regulated genes identified by the genome-wide microarray analysis on FACS-sorted SCs from the uninjured TA of WT-Pax7 (n=3) and KI-Pax7 (n=3) mice. Expression of genes is presented as centered and scaled log2 fluorescence intensity (red to yellow key), each row represents a gene, named by its MGI symbol. Confirmation by specific RTqPCRs for the three most up-regulated genes (B) and the three most down-regulated genes (C). Data are represented as the fold change of expression in KI SCs (n=5) compared to WT SCs (n=5) with the use of Wilcoxon signed rank test. SCs from (n=3) WT (Tg:Pax7nGFP) and (n=3) KI (CXCL12Gagtm/Gagtm::Pax7nGFP) were sorted by FACS and plated to assess their behavior by live videomicroscopy: (D) the velocity, (E) the onset first cell division and (F) the division rate. (n=100 cells counted). (G) Quantification by immunostaining 2 days post plating of percentage of MyoD and Pax7 population in WT vs KI SCs and 4 days post plating of percentage of Myogenin and Pax7 population in WT vs KI SCs (n=3 mice per condition and per time point). Data are given as the mean ± SEM. * p < 0.05; ** p < 0.01. [file 13395_2019_210_MOESM3_ESM.docx]

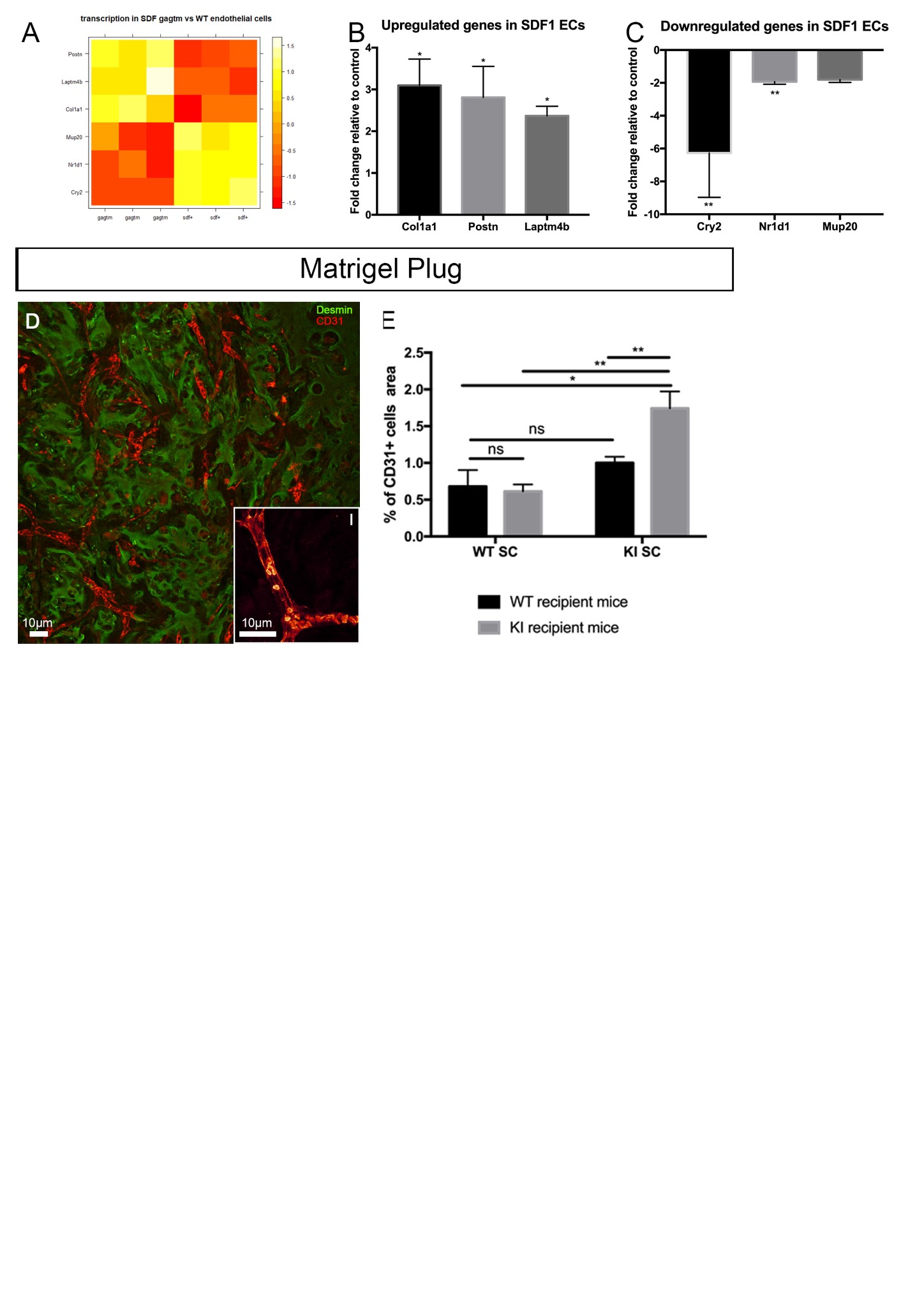

Supplement: Supplementary file 4 — Additional file 4: Figure S3. (Related to Figure 2). KI ECs display an increased angiogenic response. (A) Heat map of the three most up- and down-regulated genes identified by the genome-wide microarray analysis on FACS-sorted ECs from the uninjured TA of WT-Flk1 (n=3) and KI-Flk1 (n=3) mice. Expression of genes is presented as centered and scaled log2 fluorescence intensity (red to yellow key), each row represents a gene, named by its MGI symbol. Confirmation by specific RTqPCRs for the three most up-regulated genes (B) and the three most down-regulated genes (C). Data are represented as the fold change of expression in KI ECs (n=5) compared to WT ECs (n=5) with the use of Wilcoxon signed rank test. (D) Representative immunostaining of matrigel plugs mixed with KI (CXCL12Gagtm/Gagtm::Pax7nGFP) satellite cells inserted for 3 weeks in KI (CXCL12Gagtm/Gagtm) mice with endothelial cells (CD31, red) and myoblasts (Desmin, green). Scale bars represent 10 μm. (E) Quantification of the CD31 positive/negative surface ratio in matrigel plugs mixed with the KI (CXCL12Gagtm/Gagtm::Pax7nGFP) or the WT (Tg:Pax7nGFP) SCs inserted for 3 weeks in either the WT (C57Bl6) or the KI (CXCL12Gagtm/Gagtm) mice. Data are mean percentage ± SEM (10 fields per matrigel plug). Five animals (n=5) were used per condition and were repeated independently two times. Data are given as the mean ± SEM.* p < 0.05; ** p < 0.01. [file 13395_2019_210_MOESM4_ESM.docx]

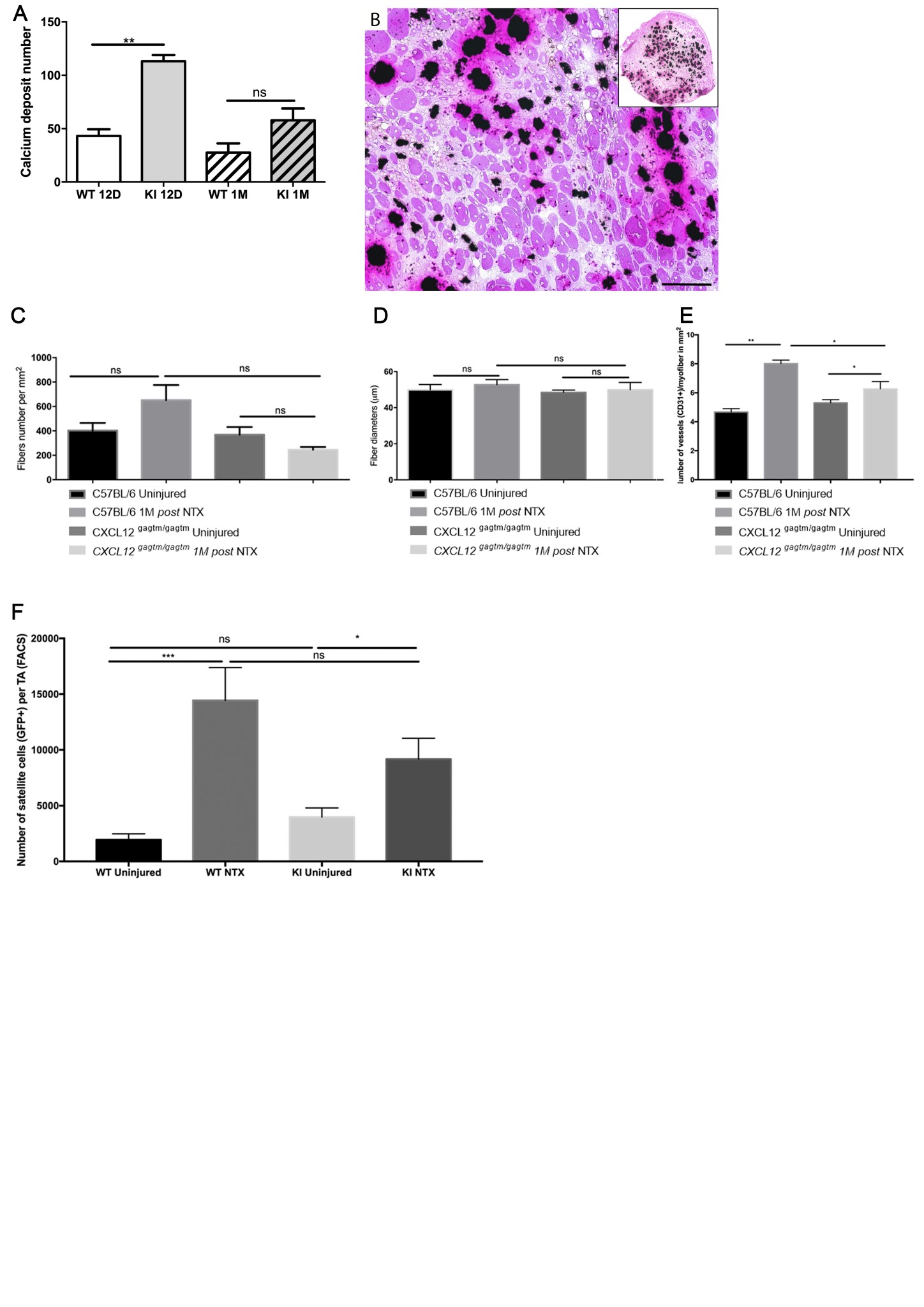

Supplement: Supplementary file 5 — Additional file 5: Figure S4. (Related to Figure 3). Quantitative histological and cytometric parameters of the muscle one month post NTX injury in the WT and the CXCL12Gagtm/Gagtm mice. (A) Quantification of calcium deposits number by Von Kossa staining in 12 days and one month post NTX injured TA from WT (C57Bl6) and CXCL12Gagtm/Gagtm mice. Three animals (n=3) were used per condition and were repeated independently two times. (B) Representative Von Kossa stained TA section 12 post NTX injury in KI (CXCL12Gagtm/Gagtm) mice. Scale bar represent 100μm. Quantification of (C) fibers number and (D) fibers diameter by Hematoxylin-eosin staining in uninjured and post NTX injured TA from WT (C57Bl6) and CXCL12Gagtm/Gagtm mice. Three animals (n=3) were used per condition and were repeated independently two times. (E) Quantification of vessels number by CD31 immunostaining in the uninjured and the post NTX injured TA from WT (C57Bl6) and CXCL12Gagtm/Gagtm mice. Three animals (n=3) were used per condition and were repeated independently two times. (F) Quantification of GFP-positive cells by FACS analysis per TA of the uninjured and the post NTX injured WT (Flk1GFP/+) vs. KI (CXCL12Gagtm/Gagtm :: Flk1GFP/+) mice. (n=5 mice per condition). Data are given as the mean ± SEM. * p < 0.05; ** p < 0.01, *** p < 0.001. [file 13395_2019_210_MOESM5_ESM.docx]

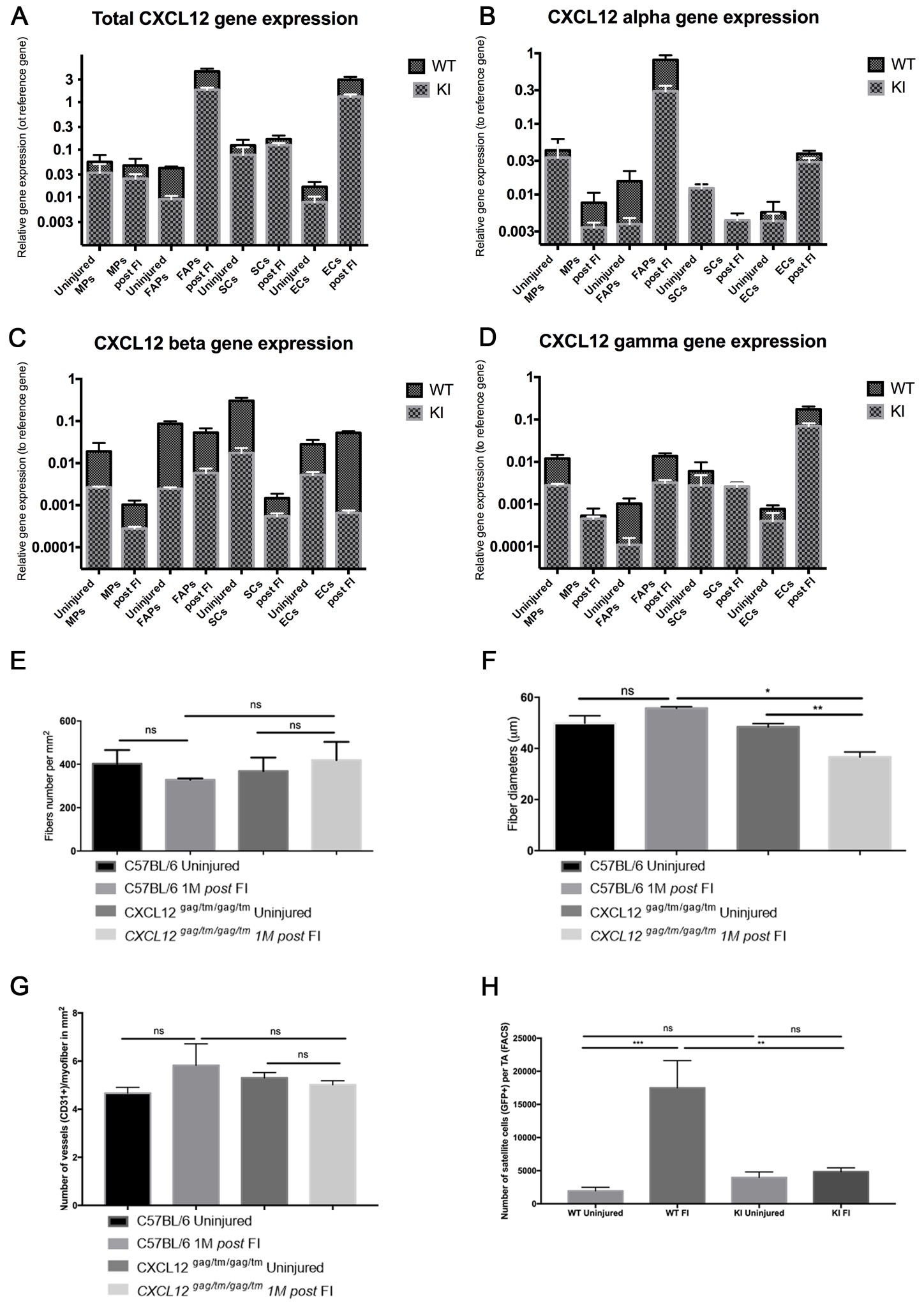

Supplement: Supplementary file 6 — Additional file 6: Figure S5. (Related to Figure 4). Quantitative histological and cytometric parameters of the muscle one month post FI in WT and CXCL12Gagtm/Gagtm mice. RTqPCR gene expression in FACS-sorted MPs, FAPs, SCs, ECs from TA muscle without injury and TA 12 days post FI from WT-Pax7 and KI-Pax7 with relative expression, represented as a log2, of total CXCL12 gene (A), alpha CXCL12 isoform gene (B), beta CXCL12 isoform gene (C) and gamma CXCL12 isoform gene (D). The data are represented in Log2 and n=3 animals per condition for uninjured mice, n=5 animals for injured WT-Pax7 mice and n=6 animals for injured KI-Pax7 mice. Quantification of (E) the number of fibers and (F) the fiber’s diameter by Hematoxylin-eosin staining in the uninjured and post FI TA from WT (C57Bl6) and CXCL12Gagtm/Gagtm mice. Three animals (n=3) were used per condition and were repeated independently two times. (G) Quantification of the number of vessels by CD31 immunostaining in the uninjured and the post FI injured TA from WT (C57Bl6) and CXCL12Gagtm/Gagtm mice. Three animals (n=3) were used per condition and were repeated independently two times. (H) Quantification of GFP-positive cells by FACS analysis per the TA of uninjured and post FI injured WT (Flk1GFP/+) vs. KI (CXCL12Gagtm/Gagtm :: Flk1GFP/+) mice. (n=5 mice per condition). Data are given as the mean ± SEM. * p < 0.05; ** p < 0.01, *** p < 0.001. [file 13395_2019_210_MOESM6_ESM.docx]

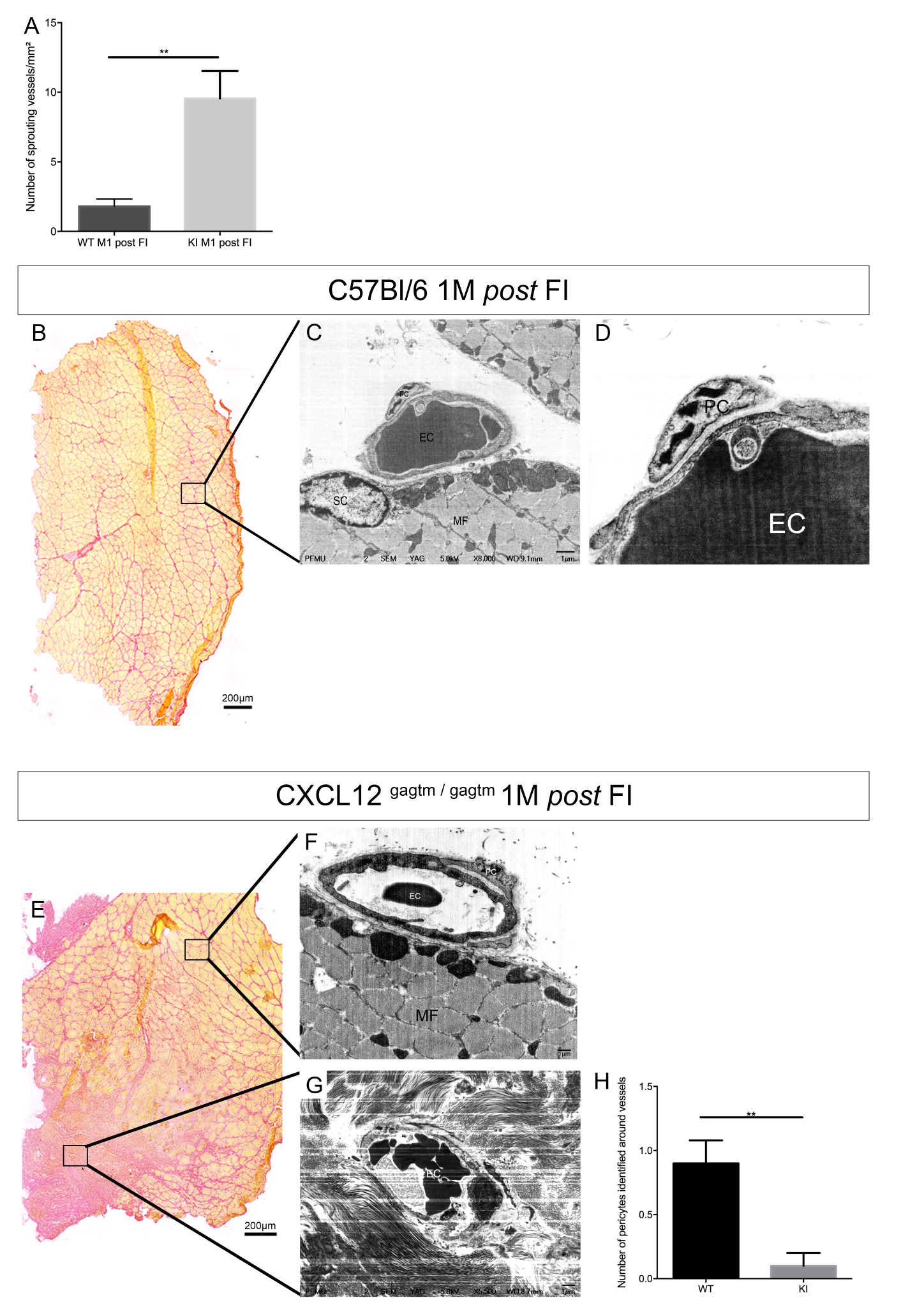

Supplement: Supplementary file 7 — Additional file 7: Figure S6. (Related to Figure 5). Quantitative histological and ultrastructure parameters of the vascular network of muscle one month post FI in WT and KI mice. (A) Quantification of the sprouting vessels number one month post FI in the muscle of WT (Flk1GFP/+) and KI (CXCL12Gagtm/Gagtm :: Flk1GFP/+) mice (n=5). (B and E) Scanning Electron Microscopy (SEM) sample were selected according to Sirius Red staining. Scale bar represent 200 μm. (C and D) A set of representative images of a satellite cell (SC); a myofiber (MF); an endothelial cell (EC); a pericyte (PC) from WT (C57Bl6) muscle. Scale bar represent 1 μm. (F-G) Representative images of a SC, a MF a EC and a PC from two different zones selected on Sirius Red section in KI (CXCL12Gagtm/Gagtm) muscles: (F) in a fully regenerated area and (G) in the non-regenerated zone. Scale bar represent 1 μm. (H) Number of pericytes identified around vessels in the non-regenerated zone of KI muscle compared to WT muscle one month post FI. Ten fileds including at least one vessel were randomly counted on each TA section for each condition (n=3 animals per condition). Data are given as the mean ± SEM. p < 0.05; ** p < 0.01. [file 13395_2019_210_MOESM7_ESM.docx]
